# Theoretical Study of Antioxidant and Prooxidant Potency of Protocatechuic Aldehyde

**DOI:** 10.3390/ijms26010404

**Published:** 2025-01-05

**Authors:** Ana Amić, Denisa Mastiľák Cagardová, Žiko Milanović

**Affiliations:** 1 Department of Chemistry, Josip Juraj Strossmayer University of Osijek, Ulica cara Hadrijana 8A, 31000 Osijek, Croatia; 2 Institute of Physical Chemistry and Chemical Physics, Department of Chemical Physics, Slovak University of Technology in Bratislava, Radlinského 9, SK-812 37 Bratislava, Slovakia; denisa.cagardova@stuba.sk; 3 Institute for Information Technologies Kragujevac, Department of Science, University of Kragujevac, Jovana Cvijića bb, 34000 Kragujevac, Serbia; ziko.milanovic@uni.kg.ac.rs

**Keywords:** protocatechuic aldehyde, DFT, antioxidant potency, radical scavenging, repair of damaged biomolecules, kinetics, iron ions chelation, molecular docking, xanthine oxidase

## Abstract

In this study, the antioxidant and prooxidant potency of protocatechuic aldehyde (PCA) was evaluated using density functional theory (DFT). The potency of direct scavenging of hydroperoxyl (HOO^•^) and lipid peroxyl radicals (modeled by vinyl peroxyl, H_2_C=CHOO^•^) involved in lipid peroxidation was estimated. The repair of oxidative damage in biomolecules (lipids, proteins and nucleic acids) and the prooxidant ability of PCA phenoxyl radicals were considered. The repairing potency of PCA was investigated for damaged tryptophan, cysteine, leucine, DNA base guanine and linolenic acid. The thermodynamics and kinetics of the single electron transfer (SET) and formal hydrogen atom transfer (fHAT) mechanisms underlying the studied processes were investigated under physiological conditions in aqueous and lipid environments using the SMD/M06-2X/6-311++G(d,p) level of theory. Sequestration of catalytic Fe^2+^ and Fe^3+^ ions by PCA, which prevents HO^•^ production via Fenton-like reactions, was modeled. Molecular docking was used to study the inhibitory capability of PCA against xanthine oxidase (XO), one of the enzymes producing reactive oxygen species. The attained results show that PCA has the capability to scavenge lipid peroxyl radicals, repair damaged tryptophan, leucine and guanine, chelate catalytic iron ions and inhibit XO. Thus, PCA could be considered a possible multifunctional antioxidant.

## 1. Introduction

Reactive species such as free radicals are constantly produced in cells to ensure normal cellular activity [1]. In very low concentrations, free radicals assist health and longevity by functioning as crucial chemical messengers [2]. Additionally, the immune system uses free radicals to resist infections. Specific cellular systems regulate the production and removal of free radicals, resulting in a delicate balance. If this balance is disrupted, high concentrations of free radicals induce a state of oxidative stress, leading to oxidative damage of macromolecules (lipids, proteins and nucleic acids) which may initiate numerous illnesses and aging [3].

Traditional knowledge and epidemiological evidence suggest that a diet based on fruit and vegetables (e.g., Mediterranean diet) contributes to a low occurrence of cardiovascular diseases and some types of cancer [4]. The positive effects of such eating behavior are partly attributed to polyphenols, compounds commonly found in many plants [5]. When endogenous protective mechanisms (cell enzymes) are unable to eliminate surplus radicals, polyphenols, as non-enzymatic antioxidants, may contribute to restoring equilibrium. The belief still exists that decreasing the surplus of free radicals and repressing their generation through dietary components could help to reduce the risk of illness occurrence. Several mechanisms underlying the health-promoting activities of polyphenols have been proposed, such as direct radical scavenging, catalytic metal sequestration and the inhibition of prooxidant enzymes [6,7].

**Figure 1:**
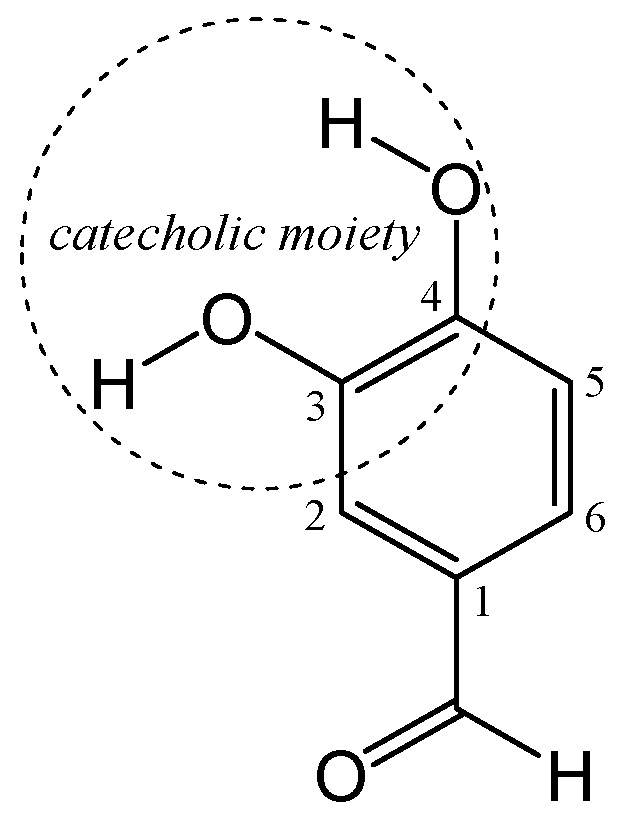
Structure, atom numeration and catecholic moiety of protocatechuic aldehyde (PCA).

Protocatechuic aldehyde (PCA) is a commonly occurring catecholic compound present in barley, green cavendish bananas, grapevine leaves, marine red algae and many herbs [8]. PCA has been a subject of continuous interest due to its beneficial effects on human health. It exerts an extensive variety of bioactivities including antioxidant [9], protection against peroxidative damage to biomembranes [10], inhibition of tyrosinase [11], anticancer activity [8,12] and cardioprotection [13]. Besides its beneficial effects, recent studies have shown that PCA can exhibit acute toxicity and cardiotoxicity at higher concentrations (70–80 µg/mL), as observed in zebrafish models [14]. Biological activities and possible underlying mechanisms could be related to the main structural feature of PCA, i.e., to its vicinal OH groups (the catecholic moiety), Figure 1.

The catecholic moiety is crucial for PCA’s ability to scavenge free radicals, chelate catalytic metal ions, bind to the active sites of prooxidant enzymes and upregulate endogenous antioxidants, thus preventing oxidative damage. PCA’s biological significance has been shown in anti-inflammatory and chemopreventive contexts [15,16]; nevertheless, its dual antioxidant and prooxidant properties under different physiological situations are not yet fully comprehended. This study utilizes sophisticated computational techniques to bridge this gap, providing a detailed theoretical insight into the thermodynamics and kinetics of PCA’s antioxidant and prooxidant behavior in physiological environments. PCA may serve as a model compound for studying phenolic antioxidants because it is both structurally simple and biologically active, allowing for detailed theoretical investigations without excessive computational complexity.

In the first part of this report, the thermodynamic and kinetic potency of PCA in scavenging hydroperoxyl, HOO^•^ and vinyl peroxyl radical, H_2_C=CHOO^•^ (the simplest model of lipid peroxyl radical, LOO^•^), was investigated. HOO^•^ and LOO^•^ are involved in lipid peroxidation, a complex sequence of reactions causing pathological changes that contribute to the development of degenerative diseases. Lipid peroxidation can be initiated by the removal of the *bis*-allylic H-atom of polyunsaturated fatty acids (PUFA) of lipids (LH) in cell membranes by free radicals (O_2_^•−^, HOO^•^, HO^•^, etc.) produced in biological systems [17]:

LH + HOO^•^ → L^•^ + HOOH (1)

L^•^ + O_2_ → LOO^•^ (2)

LOO^•^ is the chain carrier of the propagation step:

LOO^•^ + LH → LOOH + L^•^ (3)

Both the initiation step and the propagation chain reaction can be terminated by peroxyl scavengers such as phenolic compounds via the formal hydrogen atom transfer (fHAT) mechanism [18]:

(4) $$\mathrm{ArOH}+{\mathrm{LOO}}^{•}\;\overset{\;\;\;k\;\;\;}{\to }{\mathrm{ArO}}^{•}+\mathrm{LOOH}$$

In non-polar media, e.g., inside a lipid bilayer, a phenolic antioxidant with a rate constant *k* larger than 1.18–3.05 × 10^3^ M^−1^ s^−1^, i.e., the rate by which HOO^•^ damages PUFAs, can be considered an efficient suppressor of lipid peroxidation [19].

The majority of the theoretical kinetic investigations concerning the antioxidant potency of phenolics are focused on scavenging particular free radicals without consideration of the reactivity of the resulting phenoxyl radicals, as well as the potency of phenolics to repair damaged macromolecules, e.g., lipids, proteins and nucleic acids. The oxidative damage of biological macromolecules can lead to cell injury and cell death and significantly contributes to the development of diseases. Because preventing such damage is not always possible, repairing damaged macromolecules is essential to maintain regular cell function [20].

Antioxidant regeneration processes, or the repairing of damaged lipids, proteins and DNA by PCA were investigated in the second part of this report. This was carried out by employing simple units of those biological macromolecules [21,22]. For lipids, a reduced model of linoleic acid was used. It contains the key chemical reactivity characteristic of PUFAs, i.e., two allylic H atoms. fHAT restores the allylic hydrogens of damaged lipids. Regarding proteins, chemical repair of damaged amino acids (cysteine, leucine and tryptophan, considered likely targets) was studied. fHAT is involved when the most frequent lesions on cysteine and leucine are fixed, while single electron transfer (SET) repairs oxidized tryptophan. Guanine was selected to explore the oxidative damage of DNA because it is the most efficiently oxidized of the nucleobases, i.e., the one-electron oxidation of DNA often affects guanine locations. One-electron loss from guanine can be repaired by SET from the antioxidant [23].

An alternative antioxidant mechanism is connected with the capacity of natural antioxidants to chelate catalytic transition metal ions, generating stable complexes that hinder free radical formation by entrapping catalysts [24]. Thus, in the third part of this study, the ability of PCA to chelate Fe^2+^ and Fe^3+^ ions included in ^•^OH generation through Fenton and Haber-Weiss reactions was explored.

In the final part of the investigation, the inhibitory potential of PCA against the xanthine oxidase (XO) enzyme was examined. XO catalyzes the oxidation of hypoxanthine to xanthine and xanthine to uric acid, accompanied by the production of reactive oxygen species (ROS) and reactive nitrogen species (RNS) like O_2_^•−^, H_2_O_2_ and NO [25]. Increased activity of XO contributes to cellular damage and the onset of inflammatory diseases. Therefore, inhibiting XO activity has become a therapeutic target for reducing oxidative stress and associated disorders [26]. The potential of PCA to inhibit XO is investigated through evaluation of the binding affinities and interaction mechanisms between the primary acid-base forms of PCA and XO under physiological conditions using molecular docking simulations.

## 2. Results and Discussion

### 2.1. Potency of PCA to Directly Scavenge HOO^•^ and H_2_C=CHOO^•^ Radicals in Aqueous and Lipid Environments Under Physiological Conditions

The positioning of PCA in lipid bilayers can provide insights into lipid peroxidation inhibition. As an amphiphilic molecule (polar groups attached to a non-polar benzene ring) with a water solubility of 0.59 mol/L at pH = 7.4 [27] and a log P_o/w_ value of 0.80 [28], PCA is probably mainly located in the aqueous phase and could penetrate into the polar lipid headgroups region of the membrane-water interface. Penetration of PCA deeper into the lipid bilayer is achievable if the flip-flop transbilayer movement occurs, as has been proposed for α-tocopherol [29]. HOO^•^ radicals coming from the water phase may initiate lipid peroxidation inside the bilayer. Thus, the scavenging of HOO^•^ by PCA may contribute to the prevention of lipid peroxidation.

In aqueous environments, PCA presents an acid–base equilibrium with experimentally determined p*K*_a1_ = 7.21 and p*K*_a2_ = 11.8 [30]. Using these p*K*_a_ values, the estimated molar fractions (Mf) of the neutral, monoanionic and dianionic species of PCA at pH = 7.4 are 0.39227, 0.60771 and 2.42 × 10^−5^, respectively. Therefore, under physiological conditions in an aqueous solution, monoanionic species of PCA are more abundant than the neutral form, while the dianionic form is negligible. In aqueous (polar) media, both SET and fHAT mechanisms may be operative, while in lipid media, SET is nonviable because non-polar environments do not provide the necessary solvation for the ionic species involved in this mechanism.

The chemistry of HOO^•^ in water solutions is specific because deprotonation and disproportionation (dismutation) influence its reactivity [31]. In aqueous media, HOO^•^ is always in a pH-dependent equilibrium with its conjugate base superoxide, O_2_^•−^:

HOO^•^ ⇄ H^+^ + O_2_^•−^         p*K*_a_ = 4.8 (5)

At a physiological pH of 7.4, the molar fractions of HOO^•^ and O_2_^•−^ are 0.00251 and 0.99749, respectively.

In aqueous solutions, HOO^•^ and O_2_^•−^ readily disappear through spontaneous disproportionation [32]:

HOO^•^ + O_2_^•−^ + H_2_O ⇄ H_2_O_2_ + O_2_ + OH^−^ (6)

Disproportionation strongly depends on the pH due to the deprotonation equilibrium (5). The abundance of the reactants influences the kinetics and may be altered by the deprotonation and disproportionation of HOO^•^. At the p*K*_a_, the rate constant for spontaneous disproportionation is maximal, *k* = 9.7 × 10^7^ M^−1^ s^−1^. At pH > 6, *k* linearly depends on the pH: *k* = 6 × 10^12^ [H^+^] (M^−1^ s^−1^). Thus, at pH 7.4, *k* for spontaneous disproportionation is equal to 2.39 × 10^5^ M^−1^ s^−1^ [31]. If the HOO^•^ radical scavenging reaction occurs at a faster rate than the reaction (6), then the antioxidant is considered an in vitro efficient HOO^•^/O_2_^•−^ scavenger, while an antioxidant with a *k* value smaller than this threshold is inefficient in scavenging HOO^•^/O_2_^•−^. It should be noted that acid-base equilibrium and dismutation are characteristic of HOO^•^ and do not exist in the case of H_2_C=CHOO^•^.

Calculated thermodynamic and kinetic data (i.e., reaction Gibbs free energy Δ_r_*G*; Gibbs free energy of activation Δ*G*^≠^; reorganization energy λ; bond dissociation enthalpy BDE, in the unit of kcal/mol; TS imaginary frequency *ν*, in the unit of cm^−1^; Eckart tunneling correction *κ*^Eck^; TST rate constant *k*^TST^; diffusion rate constant *k*_D_; apparent rate constant *k*_app_; Eckart rate constant *k*^Eck^; and rate constants, including the molar fractions of reactants $k_{Mf}^{SET}$ and $k_{Mf}^{fHAT}$, in the unit of M^−1^ s^−1^) for SET from phenoxide monoanions of PCA to HOO^•^ and fHAT from OH groups of PCA to HOO^•^ are presented in Table 1a. By summing up the *k*_app_ × ^M^*f*_HA−_ × ^M^*f*_HOO•_ for monoanions, the $k_{Mf}^{SET}$ value was calculated and was estimated to be 3.37 × 10^3^ M^−1^ s^−1^. By summing up the *k*^Eck^ × ^M^*f*_H2A_ × ^M^*f*_HOO•_ for neutral PCA, the $k_{Mf}^{HAT}$ value is 8.02 × 10^−1^ M^−1^ s^−1^. *k*_overall_ ($k_{Mf}^{SET}$ + $k_{Mf}^{HAT}$) of 3.37 × 10^3^ M^−1^ s^−1^ is lower than that of the dismutation reaction (2.39 × 10^5^ M^−1^ s^−1^ [31,32]), indicating the inability of PCA to inactivate HOO^•^ radicals in water phase at pH = 7.4.

It should be noted that the rate constants measured in pH regions of biological interest most frequently represent composite rates of both HOO^•^ and O_2_^•−^ radical reactions [33]. In vitro, some plant phenolic antioxidants are able to scavenge superoxide, e.g., gallic acid (5.4 × 10^6^ M^−1^ s^−1^), quercetin (1.0 × 10^6^ M^−1^ s^−1^) and (−)-epicatechingallate (1.1 × 10^7^ M^−1^ s^−1^), among others [34]. The rate constant of the dismutation reaction (6) is very small compared to the rate constant for the reaction between antioxidant enzyme superoxide dismutase (SOD) and O_2_^•−^, *k* = 1.4 × 10^9^ M^−1^ s^−1^ [34]. In vivo, antioxidants may significantly contribute to the suppression of lipid peroxidation only if the rates of the reaction with HOO^•^/O_2_^•−^ approach the very fast rate at which O_2_^•−^ reacts with SOD [35].

Usually, the potency of phenolic compounds to scavenge HOO^•^/O_2_^•−^ is compared to that of Trolox (artificial vitamin E analog, soluble in water), which is commonly used as a reference antioxidant [36]. Trolox can be considered an antioxidant only if it can react with HOO^•^/O_2_^•−^ faster than the dismutation of this couple. However, because Trolox is not an efficient scavenger of the HOO^•^/O_2_^•−^ couple in aqueous environments (1.7 × 10^4^ M^−1^ s^−1^ [37]), this practice should be reconsidered. Simply, the HOO^•^/O_2_^•−^ couple disappears (or causes damage) faster than Trolox is able to react as a scavenger.

A different result emerges regarding H_2_C=CHOO^•^. H_2_C=CHOO^•^ could be used as the simplest model of lipid peroxyl radicals generated in the lipid bilayer under oxidative stress conditions. Inhibition of lipid peroxidation can be achieved by donating a hydrogen atom from PCA’s phenolic OH groups to a lipid peroxyl radical.

Inactivation of H_2_C=CHOO^•^ via fHAT in aqueous environments proceeds almost two orders of magnitude faster than inactivation of HOO^•^: $k_{overall}^{Eck}$ = 3.41 × 10^4^ M^−1^ s^−1^ (Table 1b) vs. $k_{overall}^{fHAT}$ = 8.02 × 10^2^ M^−1^ s^−1^ (Table 1a). The potency of phenoxide anions of PCA to scavenge H_2_C=CHOO^•^ via SET is much more pronounced: $k_{Mf}^{SET}$ = 2.68 × 10^8^ M^−1^ s^−1^ (Table 1b). This could be ascribed to the lower reaction barrier (Δ*G*^≠^) and absence of acid-base equilibrium in the case of H_2_C=CHOO^•^ (^M^*f*_H2C=CHOO•_ at any pH amounts to 1, i.e., the molar fraction does not affect the rate constant). Hence, PCA has the potential to contribute to the inhibition of lipid peroxidation in water (polar) environments provided that the diffusion-trapping mechanism, based on the floating lipid peroxyl radical hypothesis, is operative [38]. It is assumed that lipid peroxyl radicals diffuse to the surface of the membrane via conformational isomerization to regions of high polarity (at or near the polar aqueous region) where PCA is presumably located.

In non-polar media, i.e., inside a lipid bilayer where lipid peroxidation occurs, modeled by calculations in pentyl ethanoate, the potency of PCA in scavenging HOO^•^ is nearly one order of magnitude lower than in water and also insufficient to protect against HOO^•^ damage, $k_{overall}^{fHAT}$ = 8.30 × 10^2^ M^−1^ s^−1^ (Table 2a). To be protective, it must overwhelm the threshold of 1.18–3.05 × 10^3^ M^−1^ s^−1^ [19,39].

PCA has the potential to scavenge H_2_C=CHOO^•^ via fHAT ($k_{overall}^{Eck}$ = 2.24 × 10^4^ M^−1^ s^−1^, Table 2b) provided that it penetrates deeply into the bilayer via flip-flop transbilayer movement. The flip-flop mobility in the membrane coincides with the variable position of antioxidant active moiety (phenolic OH groups) inside the bilayer, thus scavenging the deeply buried LOO^•^ radicals [29].

The obtained results indicate that PCA is not able to suppress HOO^•^ induced lipid peroxidation but has the potency to scavenge lipid peroxyl radicals, i.e., to terminate the propagation chain reaction.

To the best of our knowledge, experimental results on the scavenging of HOO^•^ and H_2_C=CHOO^•^ radicals by PCA have not yet been published. Additionally, there are no available assay results related to our theoretical predictions in the forthcoming sections. Thus, our results could only be validated using scarce literature data related to similar subjects. Here, it should be noted that among the five natural hydroxybenzaldehydes and their acid analogs, in vitro assays indicate that PCA is more active in scavenging stable artificial DPPH and ABST radicals [40]. Such assays provide a quantitative measure of the H-atom donating abilities of these compounds, while the actual antioxidant efficacy should measure their ability to donate H-atom (electron) to naturally occurring radicals, such as the peroxyl radicals investigated here.

### 2.2. Potency of PCA to Repair Damaged Essential Biological Molecules

The results obtained in this section, referring to the repair of oxidatively damaged biomolecules, will reveal whether PCA may have the ability to restore biomolecules to their native form. This type of protection could stop, at least to some extent, permanent lesions and related illnesses [41].

#### 2.2.1. Potency of PCA to Repair Damaged Proteins

Oxidation (damaging) of proteins may occur at any amino acid residue situated in any protein region. Oxidative attacks from a large variety of ROS and RNS result in the formation of amino acid radicals in peptide environments. Proteins may reside in hydrophilic or hydrophobic zones and damage is predicted to occur in their residues exposed to those surroundings [42]. Thus, the studied damage and repair reactions of proteins were modeled using water and pentyl ethanoate as solvents. The main aim of this section of the study is to estimate kinetic data on the repair reactions of damaged tryptophan (a nitrogen-centred radical), cysteine (a sulfur-centered radical) and leucine (a carbon-centered radical) with PCA, as well as on the prooxidant ability of the resulting PCA phenoxyl radicals. For this purpose, we have used free amino acids, as it has been shown that there are no considerable differences between using free amino acids or models of protein-bonded amino acids [22].

##### Damaged Tryptophan Repair by the Phenoxide Anions of PCA

In vivo, proteins are the leading starting targets of the physiologically significant radicals and other reactive species generated under conditions of oxidative stress. Such reactions lead to the generation of amino acid radicals on the protein surface, as well as protein peroxyl radicals and hydroperoxides. All these species are reactive and can cause damage to enzymes, lipids, nucleic acids and endogenous antioxidants, bringing about conditions connected to oxidative stress. This chain of damage could be prevented by timely restoration of the protein radicals by antioxidants [43,44,45].

Tryptophan (Trp) is an essential amino acid that is an easy target for oxidation [22]. Both free and protein-bound tryptophan are targets of oxidative stress via exposure to UV radiation, ROS and RNS. Tryptophan can play a rate-limiting role in protein synthesis because its concentration in the human body is the lowest of all the amino acids [46]. These data suggest that the damage to tryptophan residues could be a crucial issue for protein function and for human health. Thus, repairing damaged Trp residues is essential for preserving a good state of health [47]. The free zwitterionic tryptophan and the tryptophan residue in the protein have similar reactivities [22,48].

**Figure 2:**
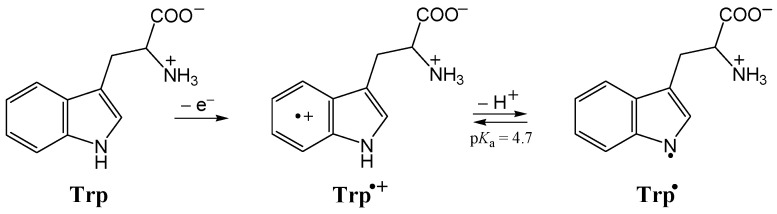
Tryptophan radical cation (Trp^•+^) produced by one-electron oxidation of tryptophan (Trp), and tryptophanyl radical (Trp^•^) produced by deprotonation of Trp^•+^.

Under oxidative stress conditions, the one-electron oxidation of tryptophan produces a tryptophan radical cation (Trp^•+^) that is rather acidic (p*K*_a_ = 4.7) [49]. At a physiological pH of 7.4, it readily deprotonates, giving a more stable neutral tryptophanyl radical (Trp^•^) [47], Figure 2.

An experimental study by Bisby et al. showed that the tryptophan radical cation is repaired faster by Trolox than the neutral tryptophanyl radical (*k* = 1.72 × 10^9^ M^−1^ s^−1^ at pH = 1.65 vs. *k* = 3.9 × 10^7^ M^−1^ s^−1^ at pH = 7.0, respectively) [50]. A theoretical study by Carreon-Gonzalez et al. revealed that thiophenols have the potential to repair damaged tryptophan via proton-coupled electron transfer (PCET) and SET mechanisms, also at close to diffusion-controlled rates [22].

It was shown by Munoz-Rugeles et al. that involvement of tryptophan radical cation (a minor species at pH = 7.4 with regard to more abundant neutral tryptophanyl radical; ^M^*f*_Trp•+_ = 0.001991 and ^M^*f*_Trp•_ = 0.9980, respectively) in the chemical repair of damaged tryptophan by uric acid is essential for the reproduction of experimental results (*k* = 1.9 × 10^7^ M^−1^ s^−1^ [44,51]) [47]. The calculated rate constant for SET from deprotonated uric acid H_2_Ur^−^ to Trp^•+^ is 2.14 × 10^6^ M^−1^ s^−1^ [47], which is one order of magnitude lower than the corresponding rate constant for SET from PCA phenoxide monoanions to Trp^•+^, 1.78 × 10^7^ M^−1^ s^−1^ (Table 3a). Thus, PCA phenoxide monoanions react via SET with the tryptophan radical cation with larger, albeit not diffusion-controlled, rates. Tryptophanyl radical repair via SET from PCA phenoxide monoanions occurs faster, with a $k_{Mf\;total}^{SET}$ rate constant of 4.55 × 10^8^ M^−1^ s^−1^. Therefore, the corresponding $k_{Mf\;overall}^{SET}$ rate constant is 4.73 × 10^8^ M^−1^ s^−1^ (Table 3b).

The *k*_overall_ rate constant calculated for reaction mechanisms involved in the repair of tryptophan radicals (Trp^•^ and Trp^•+^) by uric acid (proton–electron sequential transfer (PEST), PCET and sequential proton gain–electron transfer (SPGET)) was found to be 2.07 × 10^7^ M^−1^ s^−1^ [47]. Hence, the potency of PCA for repairing oxidatively damaged tryptophan is even higher than that of uric acid, an endogenous antioxidant which highly contributes to the plasma antioxidant capacity [52]. It should be noted that the rate constant $k_{Mf\;overall}^{SET}$ = 4.73 × 10^8^ M^−1^ s^−1^ reported in Table 3b is the lower limit, because only one kind of mechanism (i.e., SET) was investigated. For another possible mechanism in water, i.e., fHAT, the reaction of H-atom donation from PCA’s phenolic OH groups to Trp^•^ has a negative value of the Gibbs free energy of reaction (Δ_r_*G*), which means that it is thermodynamically feasible (Δ_r_*G* amounts to −6.91 kcal mol^−1^ and −6.92 kcal mol^−1^ for 3-OH and 4-OH group of PCA, respectively). Because our attempts to locate TSs failed, the related kinetics were not considered.

To predict whether repair reactions could occur, besides calculations in an aqueous medium under physiological conditions (at pH = 7.4), which represents the cell’s fluids, calculations in a lipid medium, which mimics the hydrophobic zones of proteins, i.e., the cell membrane or cavities within proteins, should also be performed. In lipid media, where the neutral species are predominant, the fHAT from the phenolic OH groups of PCA to Trp^•^ could be operative, as it is thermodynamically feasible. The Gibbs free energy of reaction, Δ_r_*G*, is exergonic: −10.29 kcal mol^−1^ and −10.65 kcal mol^−1^ for 3-OH and 4-OH group, respectively. Based on the Bell–Evans–Polanyi principle, highly exergonic reactions are predicted to be very fast. Therefore, it is expected that PCA could repair oxidative damage of tryptophan by fHAT from the catechol site. Unfortunately, we are unable to model the related kinetics because our numerous efforts to disclose the transition states for the fHAT mechanism in pentyl ethanoate as a solvent were unsuccessful.

##### Potency of PCA to Repair Damaged Cysteine

**Figure 3:**
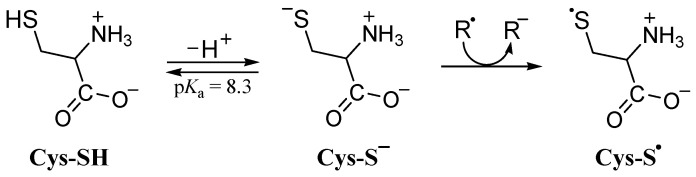
Cysteine thiol group deprotonation and thiyl radical (Cys-S^•^) formation.

Regarding protein damage, cysteine residues are among the most susceptible to oxidative damage [22]. The Thiol group of cysteine (Cys-SH) has a p*K*_a_ value of 8.3 [53], implying that in aqueous media at a physiological pH of 7.4, the molar fraction of thiolate ions (Cys-S^−^) amounts to 0.1118. It has been shown that the thiolate anion of cysteine (Cys-S^−^) is very reactive via the SET mechanism with various free radicals in polar environments (rate constants in the diffusion-limited regime), producing thiyl radicals (Cys-S^•^) and an anionic form of free radicals [54], as shown in Figure 3.

The thiyl radicals initiate a variety of damaging reactions. For example, C-centered radicals of cysteine derived from thiyl radicals could produce hydroperoxides, resulting in cell damage [55]. Consequently, antioxidants proficient in repairing thiyl radicals, i.e., restoring them to their Cys-SH or Cys-S^−^ forms, are desirable. For modeling the thermodynamics and kinetics of such processes, there are no significant differences between using free amino acids or the amino acid residue in proteins [22].

In an aqueous medium, the products of SET reactions of PCA phenoxide anions with cysteine thiyl radicals (Cys-S^•^) are PCA phenoxyl radicals and cysteine thiolate anions (repair reaction). Kinetic analysis reveals that the calculated TST rate constants are close to or in the diffusion-limited regime (*k* > 1.0 × 10^8^ M^−1^ s^−1^). More precisely, using the molar fractions of reactants, $k_{Mf\;total}^{SET}$ at pH = 7.4 is equal to 2.48 × 10^9^ M^−1^ s^−1^ (Table 4), indicating the high potency of PCA in repairing damaged cysteine.

It should be noted that under physiological conditions, numerous species can participate in competing or simultaneous reactions. Because the rate of the reverse reaction (damage reaction), i.e., the reaction between PCA phenoxyl radicals and cysteine thiolate anions producing PCA phenolate ions and cysteine thiyl radicals, is also diffusion-controlled ($k_{Mf\;total}^{SET}$ = 1.6 × 10^9^ M^−1^ s^−1^, Table 4), the potency of PCA to repair damaged cysteine in aqueous environments is reduced.

In water environments, the fHAT mechanism can be also operative, but its potency in repairing damaged cysteine by PCA, as well as in damaging cysteine by PCA phenoxyl radicals, is negligible in comparison with the SET mechanism (Table 4).

In lipid media, the repair of damaged cysteine occurs through a hydrogen transfer from phenolic -OH groups of PCA to cysteine thiyl radicals, producing PCA phenoxyl radicals and restoring -SH groups of cysteine. The data presented in Table 4 show that the repair of cysteine radicals by PCA occurs five times faster than the damage of PCA radicals to cysteine.

##### Potency of PCA to Repair Damaged Leucine

**Figure 4:**
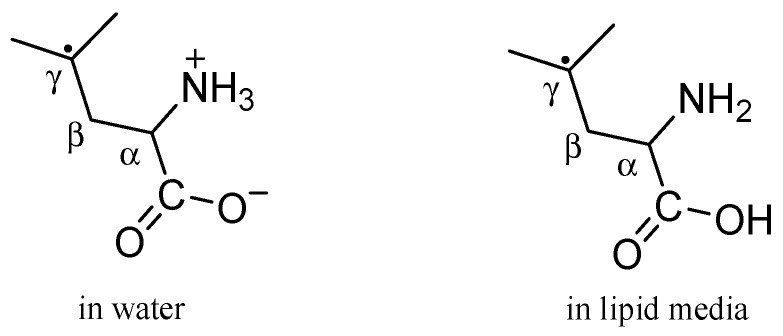
The γ-carbon-centered leucine radical.

Leucine has been recognized as a likely target of free radical damage because of its high reactivity with various biologically relevant oxidants compared to other alkyl side chain amino acids. It suffers oxidative damage mostly in the γ position of its side chain, giving rise to carbon-centered radicals (Figure 4), which are neutral and are not affected by pH [22,42]. 

In aqueous environments, reactions between γ-carbon-centered leucine radicals and PCA via SET and fHAT mechanisms were investigated to ascertain the potency of PCA to repair leucine damage. It was found that the repair of leucine radicals by PCA monoanions via the SET mechanism at pH = 7.4 (Table S1 in Supplementary Data) is highly endergonic regardless of the reaction site and consequently is not thermodynamically feasible. This is an expected result due to the low electron affinity of leucine carbon-centered radicals. Thus, PCA anions are not capable of repairing damaged leucine in aqueous environments via the SET mechanism. This result is in line with the previous finding that eight different thiophenols are not able to repair damaged leucine via SET [22].

The potency of the PCA molecule to repair damaged leucine in water at pH = 7.4 via the fHAT mechanism is much more pronounced. fHAT from both the 3-OH and 4-OH groups of PCA to γ-carbon-centered leucine radicals is exergonic and thermodynamically feasible since the leucine radical gains an H-atom and forms a stable leucine molecule. The related rate constant $k_{Mf\;total}^{fHAT}$ was estimated to be 1.02 × 10^4^ M^−1^ s^−1^ (Table 5). Phenoxyl radicals of PCA do not appear to be dangerous for leucine because their prooxidant potency is low ($k_{Mf\;total}^{fHAT}$ = 2.59 × 10^−2^ M^−1^ s^−1^, Table 5).

In comparison with thiophenols, PCA is less potent in repairing damaged leucine because the former repair at rates that are up to four orders of magnitude higher [22]. Other -SH groups containing antioxidants, e.g., glutathione and dihydrolipoic acid (with estimated apparent rate constants close to the diffusion limit), also possess high potency in leucine damage repair [56,57]. Glutathione is present at relatively high levels in most living organisms and is used by the cells as a defense against oxidative stress. Since PCA repairs damaged leucine much more slowly than glutathione, it is not likely to be physiologically relevant.

In lipid media, the fHAT mechanism is operative. The obtained results indicate that the repair of damaged leucine in the γ position by PCA via fHAT (*k* = 4.39 × 10^4^ M^−1^ s^−1^, Table 5) is nearly two times faster than that estimated for Trolox (*k* = 2.44 × 10^4^ M^−1^ s^−1^ [56]), but much slower than that predicted for dihydrolipoic acid (*k* = 4.91 × 10^7^ M^−1^ s^−1^) and glutathione (*k* = 2.27 × 10^7^ M^−1^ s^−1^) [57]. PCA phenoxyl radicals have no potency to damage leucine due to a very low related rate constant ($k_{total}^{Eck}$ = 9.01 × 10^−4^ M^−1^ s^−1^), Table 5.

#### 2.2.2. Chemical Repair of Damaged DNA by Phenoxide Anions of PCA

DNA damage is associated with numerous pathologies, such as cancer and aging. It can arise from ionizing radiation, UV light, different chemical compounds and ROS produced during metabolism. The main and transient forms of DNA damage are DNA base radicals, including radical cations and radical anions. This transient damage can cause strand breaks and generate stable base lesions [58]. Repairing the damaged sites is crucial to maintain a healthy status. Living organisms possess DNA enzymatic repair systems, which can repair DNA damage. However, the half-lives of DNA radicals are substantially shorter than the enzymatic repairing processes, which require hours [59]. Thus, the protection offered by enzymatic repair systems against DNA mutations might not be sufficient. In this case, non-enzymatic DNA repair pathways involving the fast removal of transient DNA radicals are important (non-enzymatic repair can be initiated and completed in microseconds). In cells, before the DNA enzymatic repair mechanism is initiated or when it is inhibited, DNA oxidative damage can be significantly reduced via non-enzymatic repair by natural polyphenols [60].

**Figure 5:**
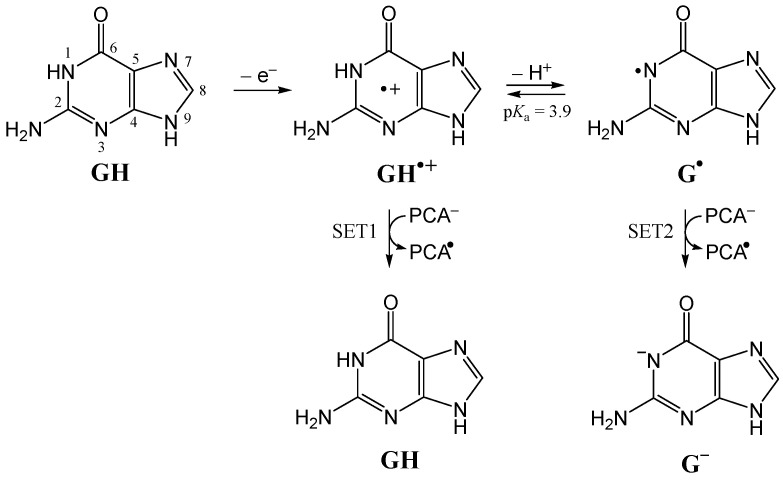
Guanine radical cation (GH^•+^) produced by one-electron oxidation of guanine (GH), and guanine radical (G^•^) produced by deprotonation of GH^•+^. Fast repair of G by SET from PCA^−^ to GH^•+^ and G^•^.

Guanine is the most susceptible DNA base to oxidation [61]. It has been determined that the guanine radical cation, G^•+^, produced by the one-electron oxidation of guanine (GH), is a Brønsted acid with a p*K*_a_ of 3.9 [62] that rapidly deprotonates to form the neutral guanine radical, G^•^, with a rate constant of 1.8 × 10^7^ s^−1^ at pH 7.0 [63]. In double-stranded DNA, deprotonation rates are lower (≥3 × 10^6^ s^−1^). It has been shown that fast non-enzymatic repair of guanine radicals of DNA (GH^•+^ and G^•^) may involve three mechanisms: PEST, SPGET and SET [64]. SET from PCA^−^ (GH^•+^ + e^−^ $\overset{SET1\;}{\to }$ GH; G^•^ + e^−^ $\overset{SET2\;}{\to }$ G^−^) would be the primary repair pathway at a physiological pH of 7.4 (Figure 5).

The results of our theoretical kinetic analysis, i.e., the estimated rate constant for G^•+^ and G^•^ repair by PCA monoanions via the SET mechanism ($k_{Mf}^{SET}$) at pH = 7.4, are presented in Table 6. At this pH, the most important reaction is the SET2 between the G^•^ and PCA^−^ (G^•^ is major fraction, ^M^*f*_G•_ = 0.9997), which contributes 99.97% to the $k_{Mf\;total}^{SET}$ and is diffusion-controlled ($k_{Mf\;total}^{SET}$ = 8.96 × 10^9^ M^−1^ s^−1^). The effective electron donation ability of the catecholic moiety (ortho-dihydroxy group) enables fast DNA repair. Due to the very low molar fraction of GH^•+^ (^M^*f*_GH•+_ = 0.000316), the contribution of SET1 is negligible. The poor prooxidant ability of PCA phenoxyl radicals (potency of electrons abstracting from guanine molecule) should be emphasized: the estimated rate constants are 1.5 × 10^−4^ M^−1^ s^−1^ and 5.3 × 10^−1^ M^−1^ s^−1^ for 3-O^•^ and 4-O^•^ PCA radical, respectively.

The results of previous experimental kinetic studies have demonstrated that numerous natural polyphenolic compounds can engage in fast electron transfer to damaged sites on DNA bases, repairing them and accordingly eliminating base damage and strand breaks [58,59,60,65,66]. It has been stated that the non-enzymatic repair pathway of polyphenolic compounds may be operative both in vitro and in vivo [59]. The chemical repair of damaged guanine by polyphenols occurs at close to diffusion-controlled rates, i.e., the repair rate constants are as high as 10^8^–10^9^ M^−1^ s^−1^ [60]. For example, using the pulse radiolytic technique, it has been estimated that the rate constant of the repair reaction of hydroxyl radical adducts of deoxyguanosine monophosphate (dGMP-OH^•^) with quercetin at pH = 7.0 is 1.31 × 10^8^ M^−1^ s^−1^ [67]. According to our results, which are summarized in Table S2, quercetin’s potency in repairing damaged free guanine is similar to that estimated for PCA. Quercetin at pH = 7.4 reacts with a two times faster rate constant with damaged guanine than PCA: 2.17 × 10^10^ M^−1^ s^−1^ vs. 8.96 × 10^9^ M^−1^ s^−1^, i.e., both reactions are controlled by diffusion.

Tan et al. suggested that the vast majority of ROS first attack DNA and that only a few ROS may be scavenged by polyphenols [59]. Polyphenols (such as quercetin), usually present at nM concentrations in plasma, cannot compete with endogenous antioxidants (such as ascorbate and urate, with μM concentrations in plasma) in scavenging ROS [52,68]. However, at low μM concentrations, they can nonenzymatically repair damaged DNA [59]. Our results indicate that this scenario could also be operative for PCA.

#### 2.2.3. Potency of PCA to Repair Damaged Lipids

**Figure 6:**
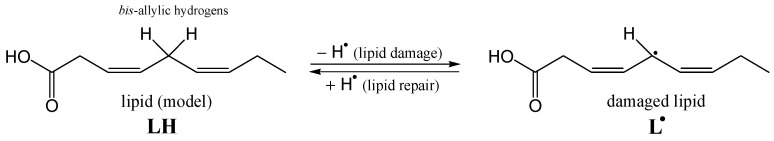
Initiation of lipid peroxidation by abstraction of the bis-allylic H-atom and repair of the damaged lipid by H-atom donation from the antioxidant.

Lipid peroxidation is one of the main occurrences in oxidative cellular damage associated with the pathogenesis of various diseases [1]. Abstraction of the *bis*-allylic H-atom of PUFA by free radicals, heat, light, metal ions, etc., initiates lipid peroxidation (Figure 6). For example, it has been experimentally determined that HOO^•^ damages linolenic acid with a rate constant of 1.70 × 10^3^ M^−1^ s^−1^ in an acidified 85% ethanolic solution [19].

To ascertain the potency of PCA to repair oxidatively damaged lipids, the lipid model was used to represent linolenic acid. This model embraces the main structural reactivity feature of PUFAs, i.e., *bis*-allylic H atoms [21]. The result of SMD/M06-2X/6-311++G(d,p) level of theory calculations in pentyl ethanoate (*k*^Eck^ = 1.9 × 10^3^ M^−1^ s^−1^, Table 7) nicely matches the experimentally determined rate constant of fHAT from linolenic acid to HOO^•^ (*k* = 1.7 × 10^3^ M^−1^ s^−1^). Using this theoretical approach, the potency of PCA to repair damaged linolenic acid and the potency of PCA phenoxyl radical to damage this PUFA were predicted. The obtained results reveal that PCA is not able to repair damaged linolenic acid (*k*^Eck^ = 1.8 × 10^−6^ M^−1^ s^−1^) and that PCA phenoxyl radicals are not harmful because they are much less reactive than HOO^•^ (Table 7).

### 2.3. Iron Chelating Ability of PCA

The aim of this part of the study is to ascertain whether PCA can act as a protector against metal-induced oxidation. Hydroxyl radicals, HO^•^, are the most dangerous radicals present in a cell. This immensely reactive specie reacts with virtually any molecule in its vicinity at or close to diffusion-controlled rates [1]. Oxidative damage of cell biomolecules by HO^•^ gives rise to various pathological conditions, such as cancer, atherosclerosis and neurodegenerative diseases [69].

Because of the remarkable ability of HO^•^ to react with any accessible molecule, it is questionable whether any antioxidant is able to directly scavenge HO^•^ [35]. In addition, direct enzymatic mechanisms for the elimination of HO^•^ do not exist [70]. Suppressing HO^•^ formation in biological systems could be a part of antioxidant therapeutic strategies [71].

It is well known that the formation of ROS (e.g., HO^•^ and O_2_^•−^) needs metal catalysis. Transition metal ions, particularly of iron, have an important role in the origin and progression of oxidative disorders, as they produce highly reactive and damaging species from rather harmless ones. For example, Fe^2+^ ion may initiate lipid peroxidation in a lipid bilayer by conversion of H_2_O_2_ into HO^•^ via a Haber–Weiss cycle (Equations (7)–(9)) [3,72]. The catalytic action of Fe^2+^ ion requires the presence of another reductant (e.g., superoxide, O_2_^•−^).

H_2_O_2_ + Fe^2+^ → HO^•^ + OH^−^ + Fe^3+^ (7)

Fe^3+^ + O_2_^•−^ → Fe^2+^ + O_2_ (8)

H_2_O_2_ + O_2_^•−^ → HO^•^ + OH^−^ + O_2_ (9)

The reaction of Fe^3+^ with O_2_^•−^ (Equation (8)) is very fast, and within the diffusion-limited regime [21]. It produces ferrous ion, Fe^2+^, thus contributing to HO^•^ formation via the Fenton reaction (Equation (7)).

To be effective as antioxidants (by suppressing the HO^•^ production), chelators must react with catalytic metal ions via exergonic sequestration reactions, i.e., stable chelates should be produced. In the aqueous environment, Fe^2+^ and Fe^3+^ ions exist as octahedral hydrated [Fe(H_2_O)_6_]^2+^ and [Fe(H_2_O)_6_]^3+^ complexes. Namely, ‘naked’ iron ions coordinate six ligands, i.e., water molecules, via the non-bonding electron pair of oxygen. Iron chelators bind to hydrated ions by replacing water molecules, making cations unable to participate in the Fenton reaction. PCA acts as a bidentate ligand towards ferrous and ferric ions. At pH = 7.4, the 4-O^−^ phenoxide anion of PCA is the dominant species (^M^*f*_HA_^−^ = 0.60771). Its catecholic oxygens are ligands in sequestration and replace two water molecules of hydrated iron ions.

Table 8 summarizes the obtained results for the chelating reactions of Fe^2+^ and Fe^3+^ ions with protocatechuic aldehyde in stoichiometric ratios of 1:1, 1:2 and 1:3. The calculated Gibbs free energies of reaction indicate that the complexation reactions of PCA in the aqueous phase at a physiological pH = 7.4 are all significantly exergonic and spontaneous in terms of thermodynamics. The 1:3 Fe^3+^−PCA complex is by far the most stable one. Lessening the reduction of Fe^3+^ ions is expected to suppress the first step of the Haber–Weiss cycle (Equation (8)), and consequently, the HO^•^ production catalyzed by Fe^2+^. Thus, PCA possesses preventive antioxidant potential via Fe^2+^ and Fe^3+^ ions chelation, in such a way as to be able to prevent their involvement in the Haber-Weiss cycle. The shown metal-chelating ability of the 4-O^−^ phenoxide anion of PCA indicates that the antioxidant potency of PCA may be partly due to its iron-chelating.

### 2.4. Inhibitory Activity of PCA Towards Xanthine Oxidase (XO)

Under normal physiological conditions, XO is present in very low amounts in serum. During an inflammatory response, it protects against infection via the generation of ROS and RNS. In the liver, XO catalyzes the oxidation of numerous metabolites, such as purines and pyrimidines, and xenobiotics, such as anti-cancer drugs [25]. During certain disease states, the levels of blood XO increase, as well as the levels of produced reactive species. O_2_^•−^ may cause further damage by reacting with H_2_O_2_ or NO and producing the very strong oxidizing agents ^•^OH and peroxynitrite (ONOO^−^). Thus, a thin line separates the benefits exerted by reactive species generated by XO from the harm they may cause. Since currently used XO inhibitors (allopurinol and febuxostat) may cause various side effects, there is a need to develop new effective and less toxic compounds. Natural polyphenols have been reported to possess a high potential for inhibiting XO [73,74].

The molecular docking study of the dominant acid–base species, PCA and PCA^−^, under physiological conditions provided significant insights into their inhibitory potential against XO. The docking simulations revealed that both the neutral and anionic forms could effectively bind to the XO enzyme active site, as evidenced by the calculated Δ*G*_bind_ and *k*_i_ values (Table 9).

The binding affinities of the compounds, represented by their Δ*G*_bind_ values, indicate moderate binding affinity, suggesting that both forms can interact favorably with the enzyme’s active site. The *K*_i_ values, which ranged from 23.4 to 24.2 μM, further corroborate these findings, highlighting the XO inhibitory potential of these compounds.

The analysis of thermodynamic parameters provided additional insights into the binding interactions. The total internal energy (Δ*G*_total_) and torsional free energy (Δ*G*_tor_) were assessed to understand the stability and flexibility of the docked complexes. The PCA exhibited slightly more favorable interaction energies compared to the PCA^−^. This can be attributed to the neutral form’s ability to form more stable hydrogen bonds and hydrophobic interactions with the enzyme’s active site, resulting in a lower Δ*G*_total_. The anionic form experiences repulsive forces due to its charge, leading to slightly higher Δ*G*_total_ values. These differences suggest that the PCA forms a more stable and energetically efficient complex with the XO enzyme, making it a potentially more effective inhibitor.

Based on the effective values of ${\Delta G}_{bind}^{eff}$ (−6.30 kcal mol^−1^) and $K_{i}^{eff}$ (23.8 μM), which indicate the total contribution of acid–base species in binding, it was determined that the binding affinity for XO is somewhat lower than that of certain flavonoids reported in the literature. For example, blumeatin (−9.200 kcal mol^−1^), quercetin (−9.993 kcal mol^−1^), kaempferol (−9.252 kcal mol^−1^), genkwanin (−9.158 kcal mol^−1^), quercetin-3-methyl ether (−8.775 kcal mol^−1^) and eriodictyol (−8.860 kcal mol^−1^) exhibit higher binding affinities [75,76]. The difference in reactivity between PCA and these flavonoids is primarily due to the presence of more aromatic rings and polar groups in flavonoids, which enhance their binding interactions with XO.

**Figure 7:**
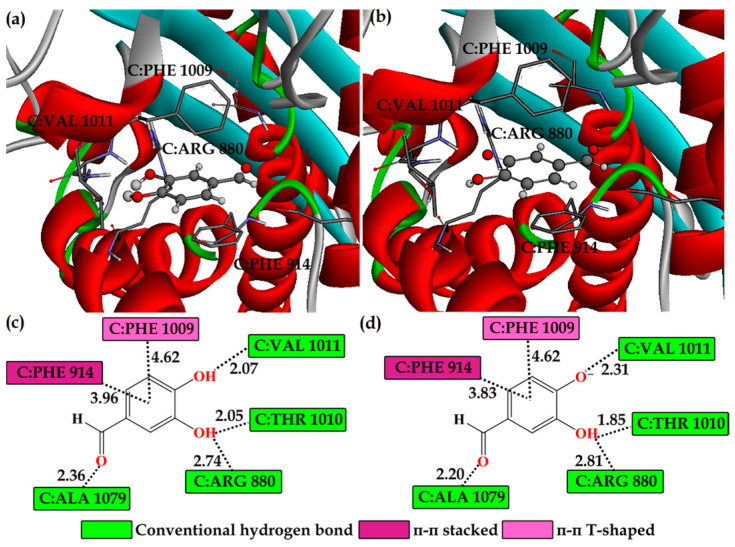
Three-dimensional (**a**,**b**) and two-dimensional (**c**,**d**) representations of interactions between acid–base species PCA (**a**,**c**) and PCA^−^ (**b**,**d**) and amino acid residues in the active site of XO (PDB Code: 3NVY), highlighting interatomic distances (Å) obtained from molecular docking simulation. Different colors indicate various types of interactions.

The visualization of the docked protein–ligand complexes using 3D and 2D representations provided a clearer understanding of the molecular interactions (Figure 7). Distance measurements between the compounds and the active site residues highlighted critical points of interaction, reinforcing the importance of these residues in the binding process. The 3D representation of interactions showcased the spatial orientation of the acid–base species within the XO active site, emphasizing the positioning of the aromatic rings in hydrophobic pockets and the alignment of hydrogen bond donors and acceptors with corresponding residues. The 2D interaction diagrams offered a detailed view of the specific interactions between the functional groups of the investigated acid–base species and the amino acid residues. This representation helped identify key residues that contribute to the binding affinity and specificity of the inhibitors. Detailed interaction analysis revealed crucial hydrogen bonds and hydrophobic interactions between the acid–base species of PCA and the amino acid residues in the XO active site.

As anticipated, the structural similarity of both acid–base species results in an identical amino acid environment within the XO active site. The main distinction resides in the distances between atoms of the acid–base species being studied and certain amino acid residues. Both species establish several conventional hydrogen bonds with crucial residues in the XO active site. Specifically, C:ALA 1079 establishes a hydrogen bond with the carbonyl oxygen of the PCA and PCA^−^. Additionally, the polar –OH group at position C3 of the acid–base species forms a bifurcated hydrogen bond with C:THR 1010 and C:ARG 880. Furthermore, C:VAL 1011 forms a shorter hydrogen bond with the -OH group of the PCA (2.07 Å) and a slightly longer bond with the PCA^−^ (2.31 Å).

These hydrogen bonds are crucial for stabilizing the inhibitor within the active site, thereby enhancing its inhibitory efficacy. Beyond conventional hydrogen bonds, the stability of the protein–ligand complexes is significantly influenced by hydrophobic interactions. The aromatic rings of the acid–base species engage in π–π interactions with hydrophobic residues C:PHE 914 (π–π stacked) and C:PHE 1009 (π–π T-shaped). These hydrophobic contacts improve binding stability by reducing solvation energy and increasing van der Waals interactions. A similar amino acid environment affects the stabilization of polyphenolic compounds such as quercetin and blumeatin [75,76].

## 3. Materials and Methods

Theoretical examination of different pathways of PCA antioxidant protection was performed following two appropriate protocols: QM-ORSA, a quantum mechanics-based test for overall free radical scavenging activity [39]; and CADMA-Chem, a computational protocol based on chemical properties and aimed at designing multifunctional antioxidants [41].

The Gaussian 09 program package [77] was used to perform geometry optimizations and frequency calculations for all structures involved in the studied radical scavenging pathways. For open-shell systems, unrestricted calculations were performed. The M06-2X functional [78], accompanied by a 6-311++G(d,p) basis set, was applied. This level of theory is able to accurately predict reaction enthalpies and barrier heights for reactions involving free radicals [39,79]. We recently confirmed the well-known fact that calculations employing the M06-2X functional are more accurate in reproducing experimentally determined rate constants than those that employ the M05-2X functional [80]. The B3LYP functional systematically underestimates BDE and Δ*G*^≠^ and should be avoided for thermodynamic and kinetic calculations [81].

The implicit solvation model SMD [82] was employed in this study to evaluate the effects of different environments in biological systems on the antioxidant and prooxidant behavior of PCA. Specifically, water was used to mimic polar, aqueous media such as cytosol, while pentyl ethanoate was selected to represent a lipid-like environment (e.g., a cellular membrane). Located transition states (TS) were confirmed as having one imaginary frequency, while local minima (reactant complex RC and product complex PC, verified by the IRC calculation) have real frequencies only. Final geometry optimization was performed on RC and PC structures. All calculations were performed at 298.15 K.

The rate constants for fHAT reactions were estimated within the framework of the conventional transition-state theory (TST) [83], as carried out in the Eyringpy program [84]:

(10) $$k=\sigma \kappa \frac{k_{B}T}{h}e^{\frac{-\left({\Delta G}^{\neq }\right)}{RT}}$$

σ is reaction path degeneracy, κ stands for tunneling corrections calculated based on the Eckart approach, *k*_B_ is the Boltzmann constant, *T* is the temperature, *h* is the Planck constant, *R* is the gas constant and Δ*G*^≠^ is the Gibbs free energy of activation of the studied reaction.

For the SET reactions, the Marcus theory [85] was applied, as carried out in the Eyringpy program [84]. It is based on the transition state formalism and enables the calculation of the barrier for any SET reaction from two thermodynamic parameters: the free energy of the reaction, ${\Delta G}_{SET}^{\neq }$, and the nuclear reorganization energy, λ:

(11) $${\Delta G}_{SET}^{\neq }=\frac{\lambda }{4}{\left(1+\frac{{\Delta G}_{SET}^{0}}{\lambda }\right)}^{2}$$

(12) $$\lambda \approx {\Delta E}_{SET}-{\Delta G}_{SET}^{0}$$

${\Delta E}_{SET}\;$ is the non-adiabatic energy difference between reactants and vertical products for SET. Accordingly, the TST rate constant for SET reactions is calculated by the Eyringpy program [84] based on the equation:

(13) $$k_{\mathrm{SET}}=\frac{k_{B}T}{h}\;e^{-({\Delta G}_{SET}^{\neq })/RT}$$

Estimated rate constants can rarely be close to the diffusion limit. In this case, the apparent rate constant (*k*_app_) and rate constant for an irreversible bimolecular diffusion-controlled reaction (*k*_D_) were calculated as implemented in the Eyringpy program [84].

For the SET mechanism, the rate constant ($k_{Mf}^{SET}$) involving the molar fractions of both reactants (antioxidant (^M^*f*_A−)_ and radical species (^M^*f*_ROO•_)) at a given pH is directly related to the experimentally determined ones under the same conditions [39,86]:

(14) $$k_{Mf}^{SET}=k_{\mathrm{app}}\times^{\;M}f_{A-}\times^{\;M}f_{\mathrm{ROO}•}$$

At a physiological pH = 7.4, ^M^*f*_HOO•_ = 0.00251, and *k*_app_ denotes the related apparent rate constant. While HOO^•^ presents an acid–base equilibrium, H_2_C=CHOO^•^ does not. Accordingly, the molar fraction of H_2_C=CHOO^•^ in aqueous solutions amounts to 1, regardless of the pH value. The molar fraction of anionic species (^M^*f*_A−_) can be determined from the related p*K*_a_ values [39].

Similarly, the rate constant $k_{Mf}^{HAT}$ for the fHAT mechanism can be computed as:

(15) $$k_{Mf}^{HAT}=k^{\mathrm{Eck}}\times^{\;M}f_{\mathrm{HA}}\times^{\;M}f_{\mathrm{ROO}•}$$

where *k*^Eck^ stands for the TST rate constant, corrected by Eckart tunneling. In an aqueous solution, both SET and fHAT can contribute to overall peroxyl scavenging. Additionally, different acid–base species can be involved in such activity. Based on the QM-ORSA protocol, which is reliable for the estimation of kinetic data that can be directly compared with the experimental values [79], the overall rate constant (*k*_overall_) has been computed by summing up the estimated rate constants for competitive SET and fHAT mechanisms:

(16) $$k_{\mathrm{overall}}=k_{Mf}^{SET}+k_{Mf}^{HAT}$$

This approach implies that once the system engages a specific pathway, it proceeds to completion, independently of the other pathway; there is no mixing or crossover between different pathways [87].

The sequestration reactions of PCA with [Fe(H_2_O)_6_]^2+^ and [Fe(H_2_O)_6_]^3+^ ions were estimated using the M06 functional because it is recommended by its developers for transition metal thermochemistry, i.e., for reactions where both organic and transition metal bonds are formed or broken [78]. The M06 functional was accompanied by the 6-311++G(d,p) basis set. This part of the investigation was performed in water as a solvent, which enables appropriate solvation for the ionic species involved. The solvation model density (SMD) was used to represent the aqueous environment [82]. The quintet (sextet) spin states of ferrous (ferric) ions were used as the most stable spin states, as proposed by Holtomo et al. [88] and Harris et al. [89], respectively.

The capacity of protocatechuic aldehyde to bind iron ions is calculated from the Gibbs free energy of chelating reactions (Δ_r_*G*) as shown for the reaction between phenoxide anion of PCA and [Fe(H_2_O)_6_]^2+^:

PCA^−^ + [Fe(H_2_O)_6_]^2+^ → [PCAFe(H_2_O)_4_]^+^ + 2 H_2_O (17)

Δ_r_*G* = *G*([PCAFe(H_2_O)_4_]^+^) + 2 × *G*(H_2_O) − *G*(PCA^−^) − *G*([Fe(H_2_O)_6_]^2+^) (18)

where *G*([PCAFe(H_2_O)_4_]^+^), *G*(H_2_O), *G*(PCA^−^) and *G*([Fe(H_2_O)_6_]^2+^) are Gibbs free energy of complex, water, PCA phenoxide anion and hydrated Fe^2+^ ion, respectively. The stability constant of iron complexes (log stability constant, log *k*; Δ_r_*G* in kJ mol^−1^) is determined using the equation [90]:

(19) $$\log\;k=\frac{{-\Delta }_{r}G}{2.303\;RT}$$

To evaluate the inhibitory effects of PCA on XO, molecular docking analysis was conducted using AutoDock Tools 1.5.7 and AutoDock 4.2.6 software [91]. This procedure consists of multiple consecutive stages, which encompass the preparation of the compounds, the selection and preparation of the protein, and the establishment of a grid for docking simulations. Firstly, the structures of the compounds were optimized using the appropriate theoretical and solvation models. The 3D X-ray crystallographic structure of the XO enzyme was acquired from the RCSB Protein Data Bank using the PDB code 3NVY (accessed 19 March 2024) [92]. The protein structures underwent processing using BIOVIA Discovery Studio 2021 [93] to eliminate any leftover heteroatoms, co-crystallized ligands and water molecules, thus ensuring a pristine and precise model for docking. The docking investigations utilized a search space for XO defined by a grid box measuring 56 × 56 × 56 Å3, with XYZ dimensions of 68.670 × 45.575 × 41.709 and a grid spacing of 0.375 Å. The Lamarckian genetic algorithm was employed for docking, treating proteins as rigid bodies and ligands as flexible entities. Key parameters included a population size of 15,027,000 generations, a maximum of 2,500,000 energy evaluations, a gene mutation rate of 0.02 and a crossover rate of 0.8. All other docking parameters were set according to established protocols from previous studies [94,95].

This study specifically analyzed the inhibitory potential of the various acid–base species of compounds. It was essential to determine the overall inhibitory activity by calculating the effective free energy of binding (${\Delta G}_{bind}^{eff}$, Equation (20)) and the effective inhibition constant ($K_{i}^{eff}$, Equation (21)) [96,97]. These values were derived by summing up the product of the molar fractions of the acid–base species (^M^*f*_PCA_/^M^*f*_PCA_^−^) and their respective binding energies (${\Delta G}_{bind}^{PCA}$/${\Delta G}_{bind}^{PCA-}$) or inhibition constants ($K_{i}^{PCA}$/$K_{i}^{PCA-}$).

(20) $${\Delta G}_{bind}^{eff}{=}^{\;M}f_{\mathrm{PCA}}\;\times \;{\Delta G}_{bind}^{PCA}{+}^{\;M}{f_{\mathrm{PCA}}}^{-}\;\times \;{\Delta G}_{bind}^{PCA-}$$

(21) $$K_{i}^{eff}\;{=}^{\;M}f_{\mathrm{PCA}}\;\times \;K_{i}^{PCA}{+}^{\;M}{f_{\mathrm{PCA}}}^{-}\;\times \;K_{i}^{PCA-}$$

## 4. Conclusions

The results presented in this study indicate that PCA has the potential to scavenge H_2_C=CHOO^•^ radicals (the simplest model of lipid peroxyl radicals) but it is ineffective in scavenging HOO^•^. Regarding the repair of oxidatively damaged biomolecules, PCA could be able to restore proteins and DNA to their genuine forms but is not able to repair damaged lipids. Phenoxyl radicals of PCA are poorly reactive and not harmful to biological macromolecules. PCA appears as a potent chelator of catalytic iron and an inhibitor of XO. In summary, PCA’s estimated antioxidant potencies indicate that this molecule could prevent, to some extent, permanent damage exerted by free radicals under oxidative stress conditions, as well as associated health disorders.

The presented approach enables a detailed understanding of the antioxidant and prooxidant behavior of PCA, providing a solid foundation for future applications in the screening of bioactive molecules across pharmaceutical, food and environmental sciences. While the approach has certain limitations, such as the simplifications of the implicit solvent model and the absence of dynamic simulations, it offers valuable insights and highlights the potential for further research combining theoretical and experimental methods. Advancements like molecular dynamics simulations and machine learning algorithms could enhance the methodology, leading to the development of predictive models with broad applicability across various scientific disciplines.

## Figures and Tables

**Table 1:** Thermodynamic and kinetic data for reaction of PCA with (a) HOO^•^ and (b) H_2_C=CHOO^•^ in water at pH = 7.40 and 298.15 K: reaction Gibbs free energy Δ_r_*G* (kcal/mol), Gibbs free energy of activation Δ*G*^≠^ (kcal/mol), reorganization energy λ (kcal/mol), TST rate constant *k*^TST^ (M^−1^ s^−1^), diffusion rate constant *k*_D_ (M^−1^ s^−1^), apparent rate constant *k*_app_ (M^−1^ s^−1^), rate constant including molar fractions of reactants $k_{Mf}^{SET}$ (M^−1^ s^−1^), bond dissociation enthalpy BDE (kcal/mol), TS imaginary frequency *ν* (cm^−1^), Eckart tunneling correction *κ*^Eck^, Eckart rate constant *k*^Eck^ (M^−1^ s^−1^) and rate constant including molar fractions of reactants $k_{Mf}^{fHAT}$ (M^−1^ s^−1^).

**(a) HOO^•^**								
SET path		Δ_r_*G*	Δ*G*^≠^	λ	*k* ^TST^	*k*_D_	*k*_app_	$k_{Mf}^{SET}$
3-O^−^		7.7	8.8	16.3	2.2 × 10^6^	7.7 × 10^9^	2.2 × 10^6^	3.36 × 10^3^
4-O^−^		11.2	11.5	15.9	2.2 × 10^4^	7.7 × 10^9^	2.2 × 10^4^	3.36 × 10^1^
$k_{Mf}^{SET}$ = 3.37 × 10^3^								
fHAT path	BDE	Δ_r_*G*	Δ*G*^≠^	*ν*	*k* ^TST^	*κ* ^Eck^	*k* ^Eck^	$k_{Mf}^{fHAT}$
3-OH	86.03	−2.5	18.1	−2582.8	3.6 × 10^−1^	2018.4	7.3 × 10^2^	7.19 × 10^−1^
4-OH	86.03	−2.5	18.8	−2555.8	1.1 × 10^−1^	793.8	8.4 × 10^1^	8.27 × 10^−2^
$k_{overall}^{fHAT}$ = 8.02 × 10^−1^								
*k*_overall_ = $k_{Mf}^{SET}$ + ${\;k}_{Mf}^{fHAT}$ = 3.37 × 10^3^ + 8.02 × 10^−1^ = 3.37 × 10^3^ M^−1^ s^−1^								
**(b) H_2_C=CHOO^•^**								
SET path		Δ_r_*G*	Δ*G*^≠^	λ	*k* ^TST^	*k*_D_	*k*_app_	$k_{Mf}^{SET}$
3-O^−^		2.8	5.6	16.6	4.5 × 10^8^	7.8 × 10^9^	4.3 × 10^8^	2.61 × 10^8^
4-O^−^		6.3	7.8	16.2	1.2 × 10^7^	7.5 × 10^9^	1.2 × 10^7^	7.29 × 10^6^
$k_{Mf}^{SET}$ = 2.68 × 10^8^								
fHAT path	BDE	Δ_r_*G*	Δ*G*^≠^	*ν*	*k* ^TST^	*κ* ^Eck^	*k* ^Eck^	$k_{Mf}^{fHAT}$
3-OH	86.03	−2.2	14.5	−2472.6	1.4 × 10^2^	534.0	7.2 × 10^4^	2.82 × 10^4^
4-OH	86.03	−2.2	16.2	−3161.9	8.4 × 10^0^	1785.7	1.5 × 10^4^	5.88 × 10^3^
$k_{overall}^{Eck}$ = 3.41 × 10^4^								
*k*_overall_ = $k_{Mf}^{SET}$ + ${\;k}_{Mf}^{fHAT}$ = 2.68 × 10^8^ + 3.41 × 10^4^ = 2.68 × 10^8^ M^−1^ s^−1^								

**Table 2:** Thermodynamic and kinetic data for reaction of PCA with (a) HOO^•^ and (b) H_2_C=CHOO^•^ via fHAT in pentyl ethanoate at 298.15 K.

**(a) HOO^•^**							
fHAT path	BDE	Δ_r_*G*	*ν*	Δ*G*^≠^	*k* ^TST^	*κ* ^Eck^	*k* ^Eck^
3-OH	84.74	−2.2	−2160.0	17.2	1.5 × 10^0^	327.6	5.1 × 10^2^
4-OH	84.37	−2.5	−2169.7	17.6	8.2 × 10^−1^	393.1	3.2 × 10^2^
$k_{overall}^{fHAT}$ = 8.30 × 10^2^							
**(b) H_2_C=CHOO^•^**							
3-OH	84.74	−1.2	−2349.1	15.3	3.6 × 10^1^	378.4	1.4 × 10^4^
4-OH	84.37	−1.5	−2350.8	15.7	2.0 × 10^1^	412.1	8.4 × 10^3^
$k_{overall}^{Eck}$ = 2.24 × 10^4^							

**Table 3:** Thermodynamic and kinetic data for the SET reaction between the PCA phenoxide monoanions and (**a**) the tryptophan radical cation, Trp^•+^, and (**b**) the tryptophanyl radical, Trp^•^, in water at pH = 7.40 and 298.15 K.

**(a) PCA^−^ + Trp^•+^**							
path	Δ_r_*G*	Δ*G*^≠^	λ	*k* ^TST^	*k*_D_	*k*_app_	$k_{Mf}^{SET}$
3-O^−^	−19.0	1.5	10.9	5.1 × 10^11^	7.4 × 10^9^	7.3 × 10^9^	8.83 × 10^6^
4-O^−^	−15.4	0.9	11.1	2.3 × 10^12^	7.4 × 10^9^	7.4 × 10^9^	8.95 × 10^6^
$k_{Mf\;total}^{SET}$ = 1.78 × 10^7^							
**(b) PCA^−^ + Trp^•^**							
3-O^−^	4.4	5.3	10.4	8.3 × 10^8^	7.4 × 10^9^	7.4 × 10^8^	4.50 × 10^8^
4-O^−^	8.0	8.1	10.0	7.7 × 10^6^	7.4 × 10^9^	7.7 × 10^6^	4.68 × 10^6^
$k_{Mf\;total}^{SET}$ = 4.55 × 10^8^							
$k_{Mf\;overall}^{SET}$ = 4.73 × 10^8^							

**Table 4:** Thermodynamic and kinetic data for the repair reactions between damaged cysteine (Cys-S^•^) with PCA species (PCA-O^−^ and PCA-OH) and damage reactions of PCA phenoxyl radicals (PCA-O^•^) with cysteine species (Cys-S^−^ and Cys-SH) via SET and fHAT mechanisms in water and pentyl ethanoate at pH = 7.40 and 298.15 K.

**SET in water**							
repair reaction: PCA-O^−^ + Cys-S^•^ → PCA-O^•^ + Cys-S^−^							
path	Δ_r_*G*	Δ*G*^≠^	λ	*k* ^TST^	*k*_D_	*k*_app_	$k_{Mf}^{SET}$
3-O^−^	3.1	3.9	8.2	8.8 × 10^9^	7.5 × 10^9^	4.0 × 10^9^	2.43 × 10^9^
4-O^−^	6.6	6.7	7.8	8.1 × 10^7^	7.4 × 10^9^	8.0 × 10^7^	4.86 × 10^7^
$k_{Mf\;total}^{SET}$ = 2.48 × 10^9^							
damage reaction: PCA-O^•^ + Cys-S^−^ → PCA-O^−^ + Cys-S^•^							
3-O^•^	−3.1	0.7	7.9	1.8 × 10^12^	7.4 × 10^9^	7.4 × 10^9^	8.3 × 10^8^
4-O^•^	−6.6	0.1	8.2	5.5 × 10^12^	7.4 × 10^9^	7.4 × 10^9^	8.3 × 10^8^
$k_{Mf\;total}^{SET}$ = 1.6 × 10^9^							
**fHAT in water**							
repair reaction: Cys-S^•^ + PCA-OH → Cys-SH + PCA-O^•^							
path	Δ_r_*G*	Δ*G*^≠^	*ν*	*k* ^TST^	*κ* ^Eck^	*k* ^Eck^	$k_{Mf}^{fHAT}$
3-OH	1.3	19.8	−1770.60	1.9 × 10^−2^	65.8	1.3 × 10^0^	5.1 × 10^−1^
4-OH	1.3	21.0	−1917.74	2.5 × 10^−3^	163.9	4.1 × 10^−1^	1.6 × 10^−1^
$k_{Mf\;total}^{fHAT}$ = 6.7 × 10^−1^							
damage reaction: PCA-O^•^ + Cys-SH → PCA-OH + Cys-S^•^							
3-O^•^	−1.3	18.5	−1770.60	1.8 × 10^−1^	65.8	1.2 × 10^1^	4.7 × 10^0^
4-O^•^	−1.3	19.7	−1917.74	2.3 × 10^−2^	163.9	3.8 × 10^0^	1.5 × 10^0^
$k_{Mf\;total}^{fHAT}$ = 6.2 × 10^0^							
**fHAT in pentyl ethanoate**							
repair reaction: Cys-S^•^ + PCA-OH → Cys-SH + PCA-O^•^							
3-OH	−0.9	17.2	−2174.84	1.5 × 10^0^	296.5	4.3 × 10^2^	
4-OH	−1.3	18.6	−2329.32	1.5 × 10^−1^	1096.6	1.6 × 10^2^	
$k_{total}^{fHAT}$ = 5.9 × 10^2^							
damage reaction: PCA-O^•^ + Cys-SH → PCA-OH + Cys-S^•^							
3-O^•^	0.9	18.2	−2174.84	3.0 × 10^−1^	269.5	8.9 × 10^1^	
4-O^•^	1.3	19.9	−2329.32	1.7 × 10^−2^	1096.6	1.8 × 10^1^	
$k_{total}^{fHAT}$ = 1.1 × 10^2^							

**Table 5:** Thermodynamic and kinetic data on the fHAT repair and damage reaction in (**a**) water at pH = 7.40 and (**b**) pentyl ethanoate at pH = 7.40 and 298.15 K.

**(a) fHAT in water**							
repair reaction: Leu-C^•^ + PCA-OH → Leu-CH + PCA-O^•^							
path	Δ_r_*G*	Δ*G*^≠^	*ν*	*k* ^TST^	*κ* ^Eck^	*k* ^Eck^	$k_{Mf}^{fHAT}$
3-OH	−7.9	14.2	−1756.4	2.3 × 10^2^	79.2	1.8 × 10^4^	7.1 × 10^3^
4-OH	−7.9	14.8	−1754.5	8.3 × 10^1^	94.0	7.8 × 10^3^	3.1 × 10^3^
$k_{Mf\;total}^{fHAT}$ = 1.02 × 10^4^							
damage reaction: PCA-O^•^ + Leu-CH → PCA-OH + Leu-C^•^							
3-O^•^	7.9	22.1	−1756.4	3.8 × 10^−4^	79.2	3.0 × 10^−2^	1.8 × 10^−2^
4-O^•^	7.9	22.7	−1754.5	1.4 × 10^−4^	94.0	1.3 × 10^−2^	7.9 × 10^−3^
$k_{Mf\;total}^{fHAT}$ = 2.59 × 10^−2^							
**(b) fHAT in pentyl ethanoate**							
repair reaction: Leu-C^•^ + PCA-OH → Leu-CH + PCA-O^•^							
3-OH	−10.5	13.5	−1825.0	7.8 × 10^2^	48.7	3.8 × 10^4^	
4-OH	−10.8	14.6	−1837.8	1.2 × 10^2^	48.5	5.9 × 10^3^	
$k_{total}^{Eck}$ = 4.39 × 10^4^							
damage reaction: PCA-O^•^ + Leu-CH → PCA-OH + Leu-C^•^							
3-O^•^	10.5	24.0	−1825.05	1.7 × 10^−5^	48.7	8.3 × 10^−4^	
4-O^•^	10.8	25.4	−1837.81	1.5 × 10^−6^	48.5	7.1 × 10^−5^	
$k_{total}^{Eck}$ = 9.01 × 10^−4^							

**Table 6:** Calculated thermodynamic and kinetic data for the SET reaction between the PCA phenoxide monoanions and (**a**) the guanine radical cation, GH^•+^, and (**b**) the guanine radical, G^•^ at pH = 7.40 and 298.15 K.

**(a) PCA^−^ + GH^•+^**							
path	Δ_r_*G*	Δ*G*^≠^	λ	*k* ^TST^	*k*_D_	*k*_app_	$k_{Mf}^{SET}$
3-O^−^	−20.9	1.9	11.5	2.4 × 10^11^	7.4 × 10^9^	7.2 × 10^9^	1.38 × 10^6^
4-O^−^	−17.4	0.9	11.1	1.4 × 10^12^	7.4 × 10^9^	7.4 × 10^9^	1.42 × 10^6^
$k_{Mf\;total}^{SET}$ = 2.80 × 10^6^							
**(b) PCA^−^ + G^•^**							
3-O^−^	−6.6	0.5	11.5	2.6 × 10^12^	7.4 × 10^9^	7.4 × 10^9^	4.50 × 10^9^
4-O^−^	−3.1	1.4	11.0	5.5 × 10^11^	7.4 × 10^9^	7.3 × 10^9^	4.43 × 10^9^
$k_{Mf\;total}^{SET}$ = 8.96 × 10^9^							

**Table 7:** Calculated thermodynamic and kinetic data for fHAT pathways: (**a**) lipid model with HOO^•^; (**b**) repair reaction, i.e., PCA (via 4-OH) with lipid model radical; (**c**) damage reaction, i.e., PCA (via 4-O^•^) with lipid model in pentyl ethanoate at pH = 7.40 and 298.15 K.

**(a) HOO^•^ + lipid model**					
Δ_r_*G*	*ν*	Δ*G*^≠^	*k* ^TST^	*κ* ^Eck^	*k* ^Eck^
−8.0	−1939.8	16.3	7.2 × 10^0^	270.5	1.9 × 10^3^
**(b) PCA 4-OH + lipid model radical**					
9.1	−1818.7	28.2	1.2 × 10^−8^	144.2	1.8 × 10^−6^
**(c) PCA 4-O^•^ + lipid model**					
−9.1	−1818.7	19.1	6.1 × 10^−2^	144.2	8.8 × 10^0^

**Table 8:** Structures of complexes and reaction Gibbs free energies (Δ_r_*G*) of the complexation reactions of Fe^2+^ and Fe^3+^ ions with PCA in stoichiometric ratios of 1:1, 1:2 and 1:3. Log stability constants (log *k*) of the complexes are included.

**1:1 Fe^2+^−PCA Complex**	**1:1 Fe^3+^−PCA Complex**
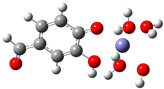	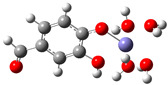
Δ_r_G = −10.92 kcal/mol	Δ_r_G = −24.92 kcal/mol
log *k* = 8.00	log *k* = 18.27
**1:2 Fe^2+^−PCA complex**	**1:2 Fe^3+^−PCA complex**
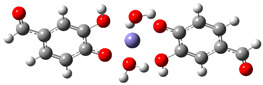	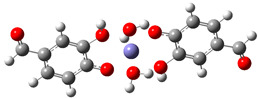
Δ_r_*G* = −19.89 kcal/mol	Δ_r_*G* = −43.02 kcal/mol
log *k* = 14.58	log *k* = 31.53
**1:3 Fe^2+^−PCA complex**	**1:3 Fe^3+^−PCA complex**
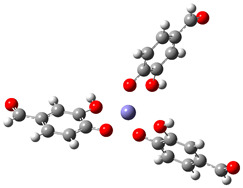	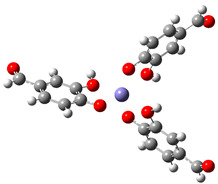
Δ_r_*G* = −31.92 kcal/mol	Δ_r_*G* = −60.68 kcal/mol
log *k* = 23.40	log *k* = 44.48

**Table 9:** Predicted thermodynamic parameters in kcal/mol: Δ*G*_bind_, Gibbs binding energy; *K*_i_, inhibition constants; Δ*G*_total_, final total internal energy; Δ*G*_tor_, torsional free energy; Δ*G*_unb_, unbound system’s energy; Δ*G*_elec_, electrostatic energy; and ${∆G}_{vdw+hbond+desolv}$, which is the sum of dispersion and repulsion (Δ*G*_vdw_), hydrogen bond (Δ*G*_hbond_) and desolvation (Δ*G*_desolv_) energy. PCA (*eff*.) represents values for the effective free energy of binding (${\Delta G}_{bind}^{eff}$) and the effective inhibition constant ($K_{i}^{eff}$) for the most favorable conformations of PCA and PCA^−^ acid–base species in the active site of XO.

Xanthine Oxidase (XO) PDB: 3NVY								
Compounds	Δ*G_bind_*	*K*_i_ (µM)	Δ*G_inter_*	Δ*G_vdw+hbond+desolv_*	Δ*G_elec_*	Δ*G_total_*	Δ*G_tor_*	Δ*G_unb_*
PCA	−6.32	23.4	−7.21	−6.92	−0.29	−1.26	0.89	−1.26
PCA^−^	−6.30	24.2	−6.89	−6.60	−0.29	−0.76	0.60	−0.76
PCA (*eff.*)	−6.30	23.8	−	–	–	–	–	–

## Data Availability

Data are contained within the article and Supplementary Materials.

## Supplementary Materials

The supporting information can be downloaded at: https://www.mdpi.com/article/10.3390/ijms26010404/s1.
