# Supplementary material for: Theoretical Study of Antioxidant and Prooxidant Potency of Protocatechuic Aldehyde
Source: Int J Mol Sci. 2025 Jan 5;26(1):404. doi: 10.3390/ijms26010404 (PMC11721355; doi:10.3390/ijms26010404)
Supplement: Supplementary file 1 [file ijms-26-00404-s001.zip › Supplementary material.pdf]

Supplementary material

Theoretical Study of Antioxidant and Prooxidant Potency of Protocatechuic Aldehyde

Ana Amić [1,*], Denisa Mastiľák Cagardová [2] and Žiko Milanović [3]

[1] Department of Chemistry, Josip Juraj Strossmayer University of Osijek, Ulica cara Hadrijana 8A, 31000 Osijek, Croatia

[2] Institute of Physical Chemistry and Chemical Physics, Department of Chemical Physics, Slovak University of Technology in Bratislava, Radlinského 9, SK-812 37 Bratislava, Slovakia; denisa.cagardova@stuba.sk

[3] University of Kragujevac, Institute for Information Technologies Kragujevac, Department of Science, Jovana Cvijića bb, 34000 Kragujevac, Serbia; ziko.milanovic@uni.kg.ac.rs

*Correspondence: aamic@kemija.unios.hr; Tel.: +381-31-399-980

Table of contents

1.	**Table S1.** Reaction Gibbs free energy $\Delta_r G$ (kcal/mol), Gibbs free energy of activation $\Delta G^{\neq}$ (kcal/mol), reorganization energy λ (kcal/mol), TST rate constant k^{TST} (M^{-1} s^{-1}), apparent rate constant k_{app} (M^{-1} s^{-1}) and rate constant including molar fractions of reactants k_{Mf}^{SET} (M^{-1} s^{-1}) for SET from PCA phenoxide monoanions to leucine radical (repair reaction of leucine radical by PCA anions in water at pH = 7.4).	3
2.	**Table S2.** Calculated data for the SET reaction between the quercetin phenoxide monoanions and: a) guanine radical cation, $GH^{\bullet+}$; b) guanine radical, $G^{\bullet}$. Reaction Gibbs free energy $\Delta_r G$ (kcal/mol), Gibbs free energy of activation $\Delta G^{\neq}$ (kcal/mol), reorganization energy λ (kcal/mol), TST rate constant k^{TST} (M^{-1} s^{-1}), diffusion rate constant k_D (M^{-1} s^{-1}), apparent rate constant k_{app} (M^{-1} s^{-1}), and rate constant including molar fractions of reactants k_{Mf}^{SET} (M^{-1} s^{-1}).	3
3.	**Table S3.** Optimized geometry and Cartesian coordinates of PCA OH···$^{\bullet}$OOH TS at M06-2X/6-311++G(d,p) level of theory in water.	4
4.	**Table S4.** Optimized geometry and Cartesian coordinates of PCA OH···$^{\bullet}$OOCH=CH_2 TS at M06-2X/6-311++G(d,p) level of theory in water.	5
5.	**Table S5.** Optimized geometry and Cartesian coordinates of PCA OH···$^{\bullet}$OOH TS at M06-2X/6-311++G(d,p) level of theory in pentyl ethanoate.	6
6.	**Table S6.** Optimized geometry and Cartesian coordinates of PCA OH···$^{\bullet}$OOCH=CH_2 TS at M06-2X/6-311++G(d,p) level of theory in pentyl ethanoate.	7
7.	**Table S7.** Optimized geometry and Cartesian coordinates of PCA OH···$^{\bullet}$S-Cys TS at M06-2X/6-311++G(d,p) level of theory in water.	8
8.	**Table S8.** Optimized geometry and Cartesian coordinates of PCA OH···$^{\bullet}$S-Cys TS at M06-2X/6-311++G(d,p) level of theory in pentyl ethanoate.	9
9.	**Table S9.** Optimized geometry and Cartesian coordinates of PCA OH···$^{\bullet}$C-Leu TS at M06-2X/6-311++G(d,p) level of theory in water.	10
10.	**Table S10.** Optimized geometry and Cartesian coordinates of PCA OH···$^{\bullet}$C-Leu TS at M06-2X/6-311++G(d,p) level of theory in pentyl ethanoate.	11
11.	**Table S11.** Optimized geometry and Cartesian coordinates of lipid model CH···$^{\bullet}$OOH TS at M06-2X/6-311++G(d,p) level of theory in pentyl ethanoate.	12
12.	**Table S12.** Optimized geometry and Cartesian coordinates of PCA 4-OH···$^{\bullet}$C lipid model radical TS at M06-2X/6-311++G(d,p) level of theory in pentyl ethanoate.	13
13.	**Table S13.** Optimized geometry and Cartesian coordinates of iron ion complexation with PCA in stoichiometric ratio 1:1 at M06/6-311++G(d,p) level of theory in water.	14
14.	**Table S14.** Optimized geometry and Cartesian coordinates of iron ion complexation with PCA in stoichiometric ratio 1:2 at M06/6-311++G(d,p) level of theory in water.	15
15.	**Table S15.** Optimized geometry and Cartesian coordinates of iron ion complexation with PCA in stoichiometric ratio 1:3 at M06/6-311++G(d,p) level of theory in water.	16
16.	**Table S16.** Optimized geometry and Cartesian coordinates of iron ions in water at M06/6-311++G(d,p) level of theory.	17

Table S1. Reaction Gibbs free energy Δ_rG (kcal/mol), Gibbs free energy of activation $\Delta G^{\neq}$ (kcal/mol), reorganization energy λ (kcal/mol), TST rate constant k^{TST} (M^{-1} s^{-1}), apparent rate constant k_{app} (M^{-1} s^{-1}) and rate constant including molar fractions of reactants k_{Mf}^{SET} (M^{-1} s^{-1}) for SET from PCA phenoxide monoanions to leucine radical (repair reaction of leucine radical by PCA anions in water at pH = 7.4).

SET path	Δ_rG	$\Delta G^{\neq}$	λ	k^{TST}	k_{app}	k_{Mf}^{SET}
3-O⁻	56.8	75.2	19.2	4.3×10^{-43}	4.3×10^{-43}	2.6×10^{-43}
4-O⁻	60.3	83.4	18.7	4.8×10^{-49}	4.8×10^{-49}	2.9×10^{-49}

Table S2. Calculated data for the SET reaction between the quercetin phenoxide monoanions and: a) guanine radical cation, $GH^{\bullet+}$; b) guanine radical, $G^{\bullet}$. Reaction Gibbs free energy Δ_rG (kcal/mol), Gibbs free energy of activation $\Delta G^{\neq}$ (kcal/mol), reorganization energy λ (kcal/mol), TST rate constant k^{TST} (M^{-1} s^{-1}), diffusion rate constant k_D (M^{-1} s^{-1}), apparent rate constant k_{app} (M^{-1} s^{-1}), and rate constant including molar fractions of reactants k_{Mf}^{SET} (M^{-1} s^{-1}).

a) quercetin + $G^{\bullet+}$							
SET path	Δ_rG	$\Delta G^{\neq}$	λ	k^{TST}	k_D	k_{app}	k_{Mf}^{SET}
3-O⁻	-26.5	4.8	11.6	2.0×10^{9}	7.5×10^{9}	1.6×10^{9}	3.39×10^{5}
5-O⁻	-15.1	0.7	9.7	1.8×10^{12}	7.5×10^{9}	7.5×10^{9}	1.59×10^{6}
7-O⁻	-11.2	0.0	10.0	5.8×10^{12}	7.5×10^{9}	7.5×10^{9}	1.59×10^{6}
3'-O⁻	-23.2	3.1	11.4	3.6×10^{10}	7.5×10^{9}	6.2×10^{9}	1.31×10^{6}
4'-O⁻	-22.6	3.6	10.4	1.4×10^{10}	7.5×10^{9}	4.9×10^{9}	1.04×10^{6}
							$k_{Mf\,total}^{SET} = 5.87 \times 10^{6}$
b) quercetin + $G^{\bullet}$							
3-O⁻	-12.2	0.0	11.6	6.1×10^{12}	7.5×10^{9}	7.5×10^{9}	5.02×10^{9}
5-O⁻	-0.8	2.1	9.7	1.9×10^{11}	7.5×10^{9}	7.2×10^{9}	4.82×10^{9}
7-O⁻	3.1	4.3	9.9	4.6×10^{9}	7.5×10^{9}	2.8×10^{9}	1.88×10^{9}
3'-O⁻	-8.9	0.1	11.4	5.0×10^{12}	7.5×10^{9}	7.5×10^{9}	5.02×10^{9}
4'-O⁻	-8.3	0.1	10.3	5.2×10^{12}	7.5×10^{9}	7.4×10^{9}	4.96×10^{9}
							$k_{Mf\,total}^{SET} = 2.17 \times 10^{10}$

Table S3. Optimized geometry and Cartesian coordinates of PCA OH···•OOH TS at M06-2X/6-311++G(d,p) level of theory in water.

PCA 3-OH				PCA 4-OH			
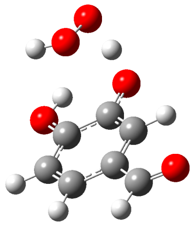				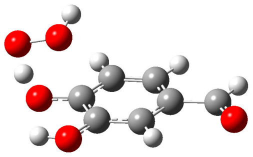			
1	2.087981000	-1.300959000	-0.577562000	1	-2.785261000	0.282118000	-0.175856000
8	2.571905000	-1.704289000	0.524523000	8	-1.956448000	-0.201398000	1.637632000
8	1.731946000	-1.148678000	1.432735000	1	-1.740564000	0.666797000	2.024202000
1	2.176765000	-0.338308000	1.739869000	8	-3.145931000	-0.038524000	1.009488000
6	-0.136376000	1.986897000	0.390760000	6	-0.138488000	1.667101000	-0.474597000
6	-1.413501000	1.476148000	0.521256000	6	1.191000000	1.557748000	-0.136793000
6	-1.739482000	0.193587000	0.038421000	6	1.784716000	0.286576000	-0.015721000
6	-0.776510000	-0.586537000	-0.580269000	6	1.060033000	-0.877939000	-0.238623000
6	0.522952000	-0.088051000	-0.736380000	6	-0.278882000	-0.778081000	-0.590115000
6	0.837229000	1.212467000	-0.236668000	6	-0.894183000	0.504322000	-0.716046000
1	-2.184807000	2.066925000	1.004495000	1	1.789414000	2.443174000	0.046631000
1	-1.008260000	-1.577618000	-0.954379000	1	1.522229000	-1.853320000	-0.141092000
6	-3.116937000	-0.299713000	0.208038000	6	3.212939000	0.216737000	0.361260000
8	-3.501791000	-1.387847000	-0.172471000	8	3.824237000	-0.821525000	0.504605000
1	-3.807802000	0.392870000	0.715900000	1	3.709483000	1.188839000	0.511337000
8	2.082461000	1.680796000	-0.366987000	8	-2.169314000	0.552967000	-1.064873000
1	2.618007000	1.035214000	-0.857403000	1	-0.629318000	2.627833000	-0.573362000
1	0.124917000	2.970889000	0.759181000	8	-1.001257000	-1.887984000	-0.805265000
8	1.481597000	-0.767207000	-1.350431000	1	-1.899088000	-1.642516000	-1.080738000

Table S4. Optimized geometry and Cartesian coordinates of PCA OH···$^{\bullet}$OOCH=CH_2 TS at M06-2X/6-311++G(d,p) level of theory in water.

PCA 3-OH				PCA 4-OH			
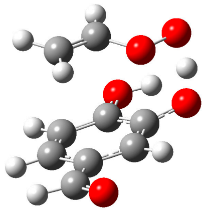				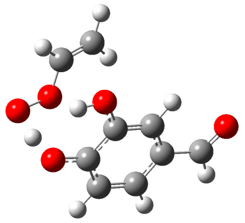			
1	-1.920227000	0.944212000	-1.221758000	1	-2.401337000	-1.221225000	-0.024208000
6	0.420594000	1.567293000	2.011366000	6	-1.228922000	2.641876000	-0.580666000
6	-0.733223000	1.574131000	1.345738000	6	-2.136615000	1.692019000	-0.365662000
1	1.340131000	1.908479000	1.550396000	1	-0.395154000	2.480664000	-1.254091000
1	0.431377000	1.234793000	3.040504000	1	-1.331600000	3.591887000	-0.074262000
1	-1.701520000	1.257081000	1.714947000	1	-3.000625000	1.751227000	0.284429000
8	-0.732991000	2.003414000	0.045511000	8	-2.006863000	0.501245000	-1.027054000
8	-1.964865000	1.975439000	-0.534700000	8	-3.000392000	-0.375395000	-0.715828000
6	-0.072249000	-1.964636000	1.000639000	6	1.248163000	0.317224000	0.806433000
6	1.226561000	-1.495921000	0.858971000	6	2.065264000	-0.268102000	-0.154443000
6	1.576929000	-0.621968000	-0.186267000	6	1.647935000	-1.401129000	-0.880807000
6	0.614937000	-0.188412000	-1.086026000	6	0.403908000	-1.944986000	-0.656851000
6	-0.714333000	-0.599770000	-0.927385000	6	-0.443944000	-1.374085000	0.312262000
6	-1.043704000	-1.515193000	0.120801000	6	0.000526000	-0.235486000	1.048797000
1	1.992446000	-1.814276000	1.558891000	1	0.049797000	-2.810427000	-1.204016000
1	0.859673000	0.506521000	-1.881429000	8	-0.817313000	0.324149000	1.954141000
6	2.963275000	-0.136401000	-0.267868000	1	-1.640386000	-0.190763000	1.998029000
8	3.368743000	0.631931000	-1.118635000	1	1.570229000	1.192439000	1.359042000
1	3.642726000	-0.513418000	0.514183000	8	-1.650802000	-1.844612000	0.572144000
8	-2.320621000	-1.895127000	0.268573000	1	2.315001000	-1.831255000	-1.619706000
1	-2.852472000	-1.468689000	-0.423810000	6	3.401960000	0.291582000	-0.440750000
1	-0.347339000	-2.637728000	1.802861000	8	3.863626000	1.264698000	0.119337000
8	-1.709955000	-0.107121000	-1.645074000	1	3.978394000	-0.236696000	-1.217018000

Table S5. Optimized geometry and Cartesian coordinates of PCA OH···$^{\bullet}$OOH TS at M06-2X/6-311++G(d,p) level of theory in pentyl ethanoate.

PCA 3-OH				PCA 4-OH			
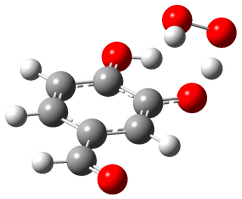				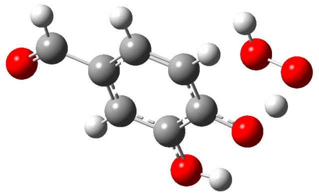			
1	2.027817000	-1.338370000	-0.611768000	1	2.813446000	-0.223279000	-0.285342000
8	1.594602000	-1.400271000	1.384958000	8	2.079719000	-0.508760000	1.603364000
1	0.749175000	-1.879616000	1.445222000	1	1.658602000	-1.386731000	1.594209000
8	2.292612000	-2.028366000	0.406560000	8	3.197349000	-0.639722000	0.848840000
6	-0.141544000	2.051175000	0.508580000	6	0.276666000	-1.285013000	-0.950384000
6	-1.406781000	1.494261000	0.565792000	6	-1.050273000	-1.422826000	-0.606350000
6	-1.677706000	0.232945000	0.005470000	6	-1.764092000	-0.320580000	-0.100954000
6	-0.669692000	-0.479297000	-0.626207000	6	-1.161902000	0.921868000	0.063279000
6	0.621609000	0.061369000	-0.700214000	6	0.174572000	1.071660000	-0.278775000
6	0.878216000	1.341058000	-0.118933000	6	0.911302000	-0.036847000	-0.793624000
1	-2.210737000	2.036984000	1.053228000	1	-1.553594000	-2.376079000	-0.725712000
1	-0.866077000	-1.449126000	-1.072308000	1	-1.722846000	1.761905000	0.456669000
6	-3.046327000	-0.319089000	0.103397000	6	-3.193614000	-0.504283000	0.256243000
8	-3.373477000	-1.403437000	-0.314524000	8	-3.898721000	0.373840000	0.687011000
1	-3.779801000	0.340599000	0.602645000	1	-3.588391000	-1.524329000	0.098827000
8	2.106780000	1.846814000	-0.191378000	8	2.173369000	0.168656000	-1.112310000
1	2.662703000	1.223492000	-0.686631000	1	0.855174000	-2.109740000	-1.351586000
1	0.073418000	3.019917000	0.942298000	8	0.784067000	2.250064000	-0.135359000
8	1.629090000	-0.545791000	-1.298117000	1	1.695392000	2.161758000	-0.456044000

Table S6. Optimized geometry and Cartesian coordinates of PCA OH···•OOCH=CH2 TS at M06-2X/6-311++G(d,p) level of theory in pentyl ethanoate.

PCA 3-OH				PCA 4-OH			
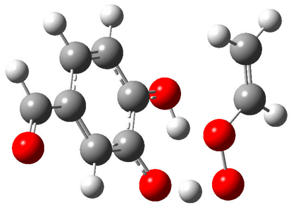				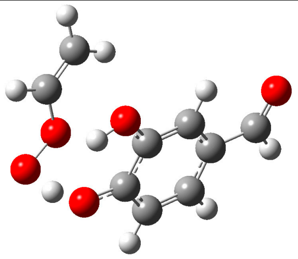			
1	1.377792000	-1.495250000	-1.218144000	1	-2.370270000	-1.268642000	0.005178000
6	2.272324000	0.441403000	2.193419000	6	-1.459700000	2.566795000	-0.568696000
6	2.511562000	-0.384421000	1.180365000	6	-2.298969000	1.539075000	-0.477524000
1	1.323732000	0.436996000	2.717235000	1	-0.539869000	2.500709000	-1.137997000
1	3.044417000	1.131892000	2.504167000	1	-1.700776000	3.489958000	-0.059608000
1	3.429427000	-0.449867000	0.607638000	1	-3.232724000	1.519438000	0.072094000
8	1.538604000	-1.261947000	0.787678000	8	-2.003283000	0.373907000	-1.123342000
8	1.940759000	-2.038235000	-0.252288000	8	-2.929183000	-0.590555000	-0.881670000
6	-0.415691000	2.043496000	0.046517000	6	1.364892000	0.361015000	0.814006000
6	-1.617040000	1.518848000	0.491895000	6	2.075964000	-0.274769000	-0.199429000
6	-2.024900000	0.220236000	0.134199000	6	1.548200000	-1.389331000	-0.877875000
6	-1.219837000	-0.566880000	-0.673645000	6	0.298907000	-1.867464000	-0.551660000
6	0.005563000	-0.063309000	-1.134129000	6	-0.446591000	-1.237031000	0.464013000
6	0.395609000	1.259796000	-0.765902000	6	0.109037000	-0.116654000	1.151043000
1	-2.260680000	2.118028000	1.128433000	1	-0.142111000	-2.716459000	-1.060875000
1	-1.518992000	-1.573449000	-0.946166000	8	-0.616556000	0.479388000	2.103064000
6	-3.317326000	-0.292425000	0.638120000	1	-1.446956000	-0.015151000	2.195014000
8	-3.759565000	-1.387095000	0.386438000	1	1.781980000	1.220803000	1.325448000
1	-3.878538000	0.409064000	1.283348000	8	-1.654942000	-1.622243000	0.816359000
8	1.571885000	1.719059000	-1.193285000	1	2.131303000	-1.861499000	-1.661555000
1	1.965155000	1.037828000	-1.763940000	6	3.415918000	0.229072000	-0.585752000
1	-0.092627000	3.039936000	0.320977000	8	3.948725000	1.184596000	-0.075896000
8	0.841907000	-0.746236000	-1.890867000	1	3.915380000	-0.334144000	-1.394584000

Table S7. Optimized geometry and Cartesian coordinates of PCA OH···$^{\bullet}$S-Cys TS at M06-2X/6-311++G(d,p) level of theory in water.

PCA 3-OH				PCA 4-OH			
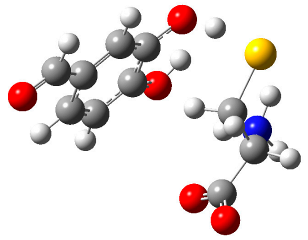				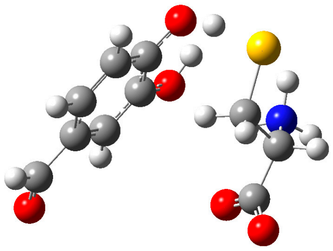			
1	0.536627000	-2.397776000	-0.210868000	1	1.279794000	-2.350965000	-0.066646000
8	-0.197106000	-2.365103000	0.673148000	8	0.359467000	-2.507012000	0.597726000
6	-0.968815000	-1.303242000	0.649585000	6	-0.649301000	-1.750567000	0.244892000
6	-0.728801000	-0.293199000	1.636097000	6	-1.056447000	-0.735176000	1.174942000
6	-1.996688000	-1.111430000	-0.296443000	6	-1.325300000	-1.887202000	-0.988999000
6	-1.495400000	0.875314000	1.660357000	6	-2.110690000	0.114690000	0.862751000
6	-2.747996000	0.041981000	-0.254144000	6	-2.365130000	-1.042630000	-1.280206000
6	-2.493278000	1.034607000	0.726418000	6	-2.753433000	-0.044540000	-0.354479000
1	-1.289265000	1.627381000	2.411425000	1	-2.410900000	0.886518000	1.561554000
8	0.247918000	-0.471964000	2.519357000	1	-2.900327000	-1.126172000	-2.219379000
1	0.638096000	-1.355640000	2.392338000	8	-0.405288000	-0.607090000	2.330931000
1	-2.169822000	-1.876798000	-1.046274000	1	0.279730000	-1.295530000	2.394985000
6	1.463368000	-0.264303000	-1.237456000	1	-0.994927000	-2.657406000	-1.675709000
1	3.803352000	0.251217000	1.129705000	6	-3.880095000	0.850467000	-0.715988000
1	2.179508000	0.170934000	1.432921000	8	-4.291157000	1.729350000	0.009594000
8	1.769560000	2.274168000	0.783366000	1	-4.334673000	0.669558000	-1.702595000
8	2.187591000	2.673241000	-1.386613000	6	1.830651000	-0.038334000	-1.001042000
6	2.161187000	1.959240000	-0.363333000	1	3.110809000	1.164119000	1.865578000
6	2.604990000	0.495465000	-0.562305000	1	1.540426000	0.665477000	1.691498000
7	2.913449000	-0.100007000	0.765544000	8	0.765855000	2.490552000	0.672009000
16	1.692412000	-2.057811000	-1.346314000	8	1.844140000	3.026417000	-1.224556000
1	3.510933000	0.448710000	-1.163661000	6	1.624079000	2.315296000	-0.222522000
1	0.522409000	-0.057623000	-0.705996000	6	2.471057000	1.030725000	-0.116896000
1	1.335039000	0.140039000	-2.242127000	7	2.488091000	0.580351000	1.300875000
1	2.958444000	-1.124536000	0.704919000	16	2.572993000	-1.685329000	-0.862905000
6	-3.823724000	0.226673000	-1.247185000	1	3.499722000	1.221152000	-0.416694000
1	-3.940357000	-0.591022000	-1.976550000	1	0.759152000	-0.105487000	-0.762371000
8	-4.546346000	1.200978000	-1.282128000	1	1.890789000	0.293911000	-2.038335000
1	-3.099699000	1.932948000	0.729811000	1	2.799416000	-0.396487000	1.368102000

Table S8. Optimized geometry and Cartesian coordinates of PCA OH···•S-Cys TS at M06-2X/6-311++G(d,p) level of theory in pentyl ethanoate.

PCA 3-OH				PCA 4-OH			
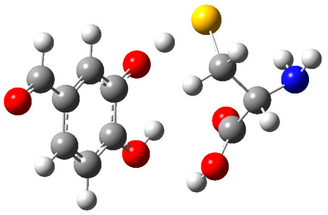				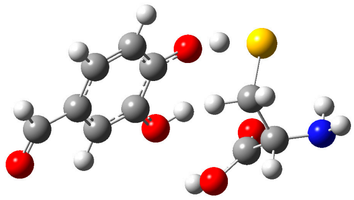			
1	0.765468000	-1.007516000	1.513497000	1	-1.268628000	1.552635000	0.999717000
7	4.093374000	-0.614628000	-0.768782000	8	-0.390018000	1.137752000	1.671650000
6	2.748869000	-0.312689000	-1.214708000	6	0.698792000	0.911217000	0.994154000
1	2.752836000	-0.168608000	-2.297186000	6	1.250489000	-0.409476000	1.069901000
6	2.301479000	0.983249000	-0.580743000	6	1.358466000	1.900722000	0.227245000
8	2.563580000	1.320178000	0.547078000	6	2.438025000	-0.708242000	0.418415000
6	1.667242000	-1.384445000	-0.905817000	6	2.523945000	1.584364000	-0.425558000
1	0.663868000	-1.000412000	-1.126575000	6	3.063565000	0.283903000	-0.325790000
1	1.830116000	-2.229727000	-1.581231000	1	2.860844000	-1.704493000	0.481145000
16	1.717034000	-2.079130000	0.768550000	1	3.043434000	2.330158000	-1.017360000
1	4.394496000	-1.499255000	-1.167931000	8	0.614513000	-1.346363000	1.778465000
1	4.091100000	-0.732240000	0.241576000	1	-0.246451000	-0.999149000	2.078522000
8	1.513889000	1.699851000	-1.391105000	1	0.922421000	2.891575000	0.177744000
1	1.189566000	2.471927000	-0.899228000	6	4.327575000	-0.016832000	-1.047646000
8	0.096143000	-0.129366000	1.900610000	8	4.865694000	-1.095270000	-1.034593000
6	-0.899491000	0.144747000	1.099972000	1	4.756178000	0.829607000	-1.613388000
6	-0.998776000	1.485082000	0.613721000	7	-4.041750000	-0.748078000	-0.890448000
6	-1.886203000	-0.793560000	0.727771000	6	-2.623522000	-0.739292000	-1.185839000
6	-2.073730000	1.872238000	-0.189437000	1	-2.446680000	-1.273963000	-2.121532000
6	-2.930021000	-0.401299000	-0.082150000	6	-1.889659000	-1.471764000	-0.087271000
6	-3.024356000	0.936189000	-0.533683000	8	-2.153991000	-1.381353000	1.085999000
1	-2.135688000	2.897938000	-0.532140000	6	-1.964061000	0.659639000	-1.333440000
8	-0.062613000	2.372794000	0.942098000	1	-0.872239000	0.572542000	-1.393346000
1	0.656785000	1.927808000	1.428988000	1	-2.293006000	1.085896000	-2.285956000
1	-1.805213000	-1.812892000	1.092953000	16	-2.424166000	1.873387000	-0.067715000
6	-3.964714000	-1.390545000	-0.463478000	1	-4.543507000	-0.240586000	-1.613813000
1	-3.804776000	-2.411493000	-0.072478000	1	-4.203714000	-0.253578000	-0.016405000
8	-4.923847000	-1.127679000	-1.146544000	8	-0.856966000	-2.183770000	-0.550407000
1	-3.863727000	1.212936000	-1.161343000	1	-0.365296000	-2.541684000	0.207313000

Table S9. Optimized geometry and Cartesian coordinates of PCA OH···$^{\bullet}$C-Leu TS at M06-2X/6-311++G(d,p) level of theory in water.

PCA 3-OH				PCA 4-OH			
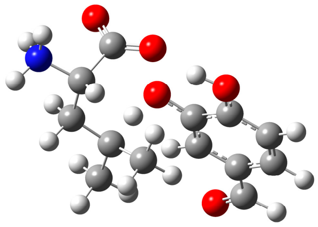				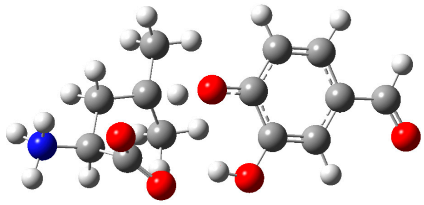			
1	0.637021000	0.530967000	-0.192116000	1	0.884413000	0.635440000	-0.363920000
7	4.818394000	0.622847000	-0.190220000	7	4.962721000	-0.126060000	0.219150000
1	4.747783000	0.893059000	-1.178045000	1	5.068397000	-0.029999000	-0.797474000
1	5.493659000	-0.144087000	-0.124310000	1	5.436143000	-0.988480000	0.502763000
1	5.178166000	1.418303000	0.345381000	1	5.425655000	0.663737000	0.678433000
6	3.470277000	0.205111000	0.303165000	6	3.505530000	-0.175908000	0.552135000
1	3.610165000	-0.221127000	1.294432000	1	3.424095000	-0.466517000	1.597193000
6	2.591181000	1.454718000	0.333557000	6	2.923914000	1.214897000	0.297536000
1	3.041817000	2.170681000	1.036381000	1	3.442189000	1.928695000	0.954644000
1	2.614096000	1.919764000	-0.658348000	1	3.149295000	1.504861000	-0.734940000
6	1.149158000	1.238088000	0.750190000	6	1.434365000	1.354757000	0.544310000
6	0.917620000	0.439328000	2.010891000	6	0.904446000	0.813851000	1.850508000
6	0.324995000	2.501242000	0.669513000	6	0.909440000	2.728312000	0.201082000
1	1.278193000	1.016620000	2.873282000	1	1.274541000	1.441149000	2.672983000
1	-0.151377000	0.261698000	2.163373000	1	-0.188514000	0.862562000	1.870680000
1	1.435399000	-0.520604000	2.004028000	1	1.212043000	-0.214671000	2.039831000
1	0.438704000	2.993599000	-0.298978000	1	1.230682000	3.046676000	-0.793185000
1	-0.734144000	2.289424000	0.839417000	1	-0.182827000	2.751901000	0.246281000
1	0.650743000	3.202732000	1.448601000	1	1.286264000	3.456959000	0.930588000
6	2.921275000	-0.858305000	-0.668070000	6	2.858989000	-1.239385000	-0.358701000
8	2.271203000	-1.801070000	-0.154419000	8	1.958145000	-1.946406000	0.153385000
8	3.154197000	-0.674552000	-1.879352000	8	3.280291000	-1.285271000	-1.532052000
6	-2.585010000	-1.944509000	0.769320000	6	-1.981805000	1.077879000	-1.377840000
6	-3.694399000	-1.140613000	0.566742000	6	-3.316290000	1.046167000	-1.014232000
6	-3.591352000	0.049001000	-0.166790000	6	-3.770690000	0.048442000	-0.144270000
6	-2.361922000	0.430914000	-0.704011000	6	-2.899601000	-0.919580000	0.361693000
6	-1.239916000	-0.373734000	-0.528569000	6	-1.564142000	-0.897693000	-0.003916000
6	-1.353479000	-1.568087000	0.234961000	6	-1.095102000	0.104153000	-0.895518000
1	-4.655181000	-1.427968000	0.981395000	1	-4.012592000	1.785554000	-1.394400000
1	-2.264587000	1.349371000	-1.273412000	1	-3.259150000	-1.686453000	1.038579000
6	-4.787171000	0.877056000	-0.347764000	6	-5.193647000	0.042282000	0.232597000
8	-4.811135000	1.934145000	-0.953533000	8	-5.700447000	-0.771051000	0.981919000
1	-5.707512000	0.481549000	0.113436000	1	-5.803425000	0.843811000	-0.215828000
8	-0.284407000	-2.345705000	0.452510000	8	0.182773000	0.085252000	-1.267403000
1	0.545453000	-1.961668000	0.084195000	1	-1.592107000	1.835121000	-2.048542000
1	-2.648635000	-2.862089000	1.341593000	8	-0.724385000	-1.830367000	0.491383000
8	-0.055616000	-0.064245000	-1.066152000	1	0.202401000	-1.694225000	0.192109000

Table S10. Optimized geometry and Cartesian coordinates of PCA OH···•C-Leu TS at M06-2X/6-311++G(d,p) level of theory in pentyl ethanoate.

PCA 3-OH				PCA 4-OH			
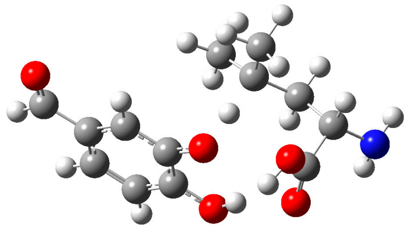				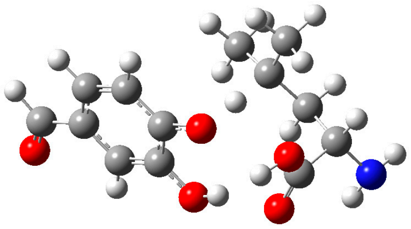			
1	0.615449000	0.732966000	0.312215000	1	0.949476000	0.868589000	0.399421000
7	4.475179000	-0.537529000	-0.913359000	6	-1.919654000	1.283110000	1.120464000
1	4.052635000	-1.385095000	-1.284477000	6	-3.235894000	1.178690000	0.706926000
1	4.852124000	-0.022722000	-1.703486000	6	-3.659199000	0.030822000	0.027263000
6	3.453226000	0.265913000	-0.252975000	6	-2.775353000	-1.016855000	-0.233797000
1	3.925914000	1.143181000	0.190234000	6	-1.455969000	-0.919902000	0.179160000
6	2.306023000	0.699209000	-1.190306000	6	-1.015050000	0.243039000	0.865246000
1	2.759410000	1.299712000	-1.992992000	1	-3.941707000	1.978723000	0.903371000
1	1.897586000	-0.200775000	-1.664951000	1	-3.114892000	-1.903118000	-0.757874000
6	1.177484000	1.510566000	-0.578249000	6	-5.069761000	-0.059053000	-0.408516000
6	1.585800000	2.729555000	0.217476000	8	-5.547575000	-1.002851000	-0.991806000
6	0.041378000	1.748805000	-1.544014000	1	-5.690537000	0.821643000	-0.159371000
1	2.099138000	3.443420000	-0.442008000	8	0.254228000	0.301826000	1.257241000
1	0.708873000	3.231861000	0.632008000	1	-1.556799000	2.158858000	1.646993000
1	2.260282000	2.486856000	1.040090000	8	-0.605003000	-1.925056000	-0.080379000
1	-0.797081000	2.258559000	-1.063156000	1	0.263920000	-1.757526000	0.323960000
1	0.387361000	2.387777000	-2.368436000	7	4.112889000	-1.404578000	-1.284248000
1	-0.321678000	0.814464000	-1.983203000	1	3.428841000	-2.112073000	-1.541389000
6	2.848303000	-0.568669000	0.854545000	1	4.488523000	-1.024581000	-2.148069000
8	2.686722000	0.100504000	1.990259000	6	3.455267000	-0.343837000	-0.530741000
8	2.494498000	-1.715211000	0.705322000	1	4.208317000	0.373595000	-0.201732000
1	2.143865000	-0.437431000	2.590041000	6	2.340979000	0.376429000	-1.320505000
6	-2.375786000	-2.093468000	-0.767059000	1	2.816899000	0.830619000	-2.202443000
6	-3.518213000	-1.315569000	-0.665700000	1	1.646486000	-0.384146000	-1.696131000
6	-3.494301000	-0.092356000	0.015145000	6	1.566288000	1.463829000	-0.596411000
6	-2.310822000	0.352590000	0.600329000	6	2.391726000	2.537536000	0.074663000
6	-1.150728000	-0.413591000	0.511223000	6	0.402261000	1.985582000	-1.403562000
6	-1.188488000	-1.651093000	-0.186742000	1	2.951904000	3.096007000	-0.688680000
1	-4.444612000	-1.656661000	-1.117635000	1	1.750209000	3.248252000	0.600564000
1	-2.282489000	1.298190000	1.131634000	1	3.110409000	2.131448000	0.788087000
6	-4.725367000	0.713066000	0.098372000	1	-0.184269000	2.715958000	-0.841144000
8	-4.808738000	1.787864000	0.646892000	1	0.779158000	2.487013000	-2.305842000
1	-5.609462000	0.263046000	-0.391659000	1	-0.261491000	1.179316000	-1.731185000
8	-0.090249000	-2.405016000	-0.294798000	6	2.817822000	-0.969795000	0.689826000
1	0.657580000	-2.007636000	0.187032000	8	2.995665000	-0.261814000	1.800050000
1	-2.381310000	-3.042022000	-1.290252000	8	2.162461000	-1.984801000	0.650740000
8	0.000156000	-0.037481000	1.069856000	1	2.426332000	-0.632536000	2.494346000

Table S11. Optimized geometry and Cartesian coordinates of lipid model CH···•OOH TS at M06-2X/6-311++G(d,p) level of theory in pentyl ethanoate.

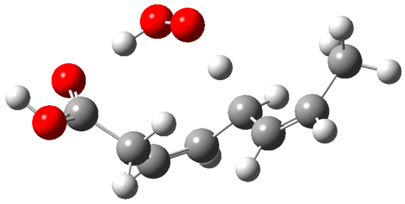

1	-0.936553000	0.858953000	0.005772000
8	0.507646000	2.404048000	0.104022000
1	1.339236000	1.895021000	0.186657000
8	-0.242748000	1.650670000	-0.752627000
6	0.860708000	-0.671728000	1.500623000
6	1.271895000	-1.174230000	0.146060000
6	-0.319633000	-0.121810000	1.809620000
6	-1.444524000	0.097052000	0.871659000
6	-1.989850000	-1.064316000	0.133402000
6	-3.095485000	-1.047004000	-0.625145000
6	-3.971548000	0.135690000	-0.889903000
1	1.615029000	-0.754255000	2.278514000
1	-0.447994000	0.251590000	2.821773000
1	-2.212474000	0.748704000	1.288684000
1	-1.438269000	-1.997347000	0.216135000
1	-3.389947000	-1.976036000	-1.107237000
1	-3.659346000	1.026674000	-0.344697000
1	-5.009501000	-0.089533000	-0.626535000
1	-3.965438000	0.376777000	-1.957872000
1	0.483415000	-1.046403000	-0.602199000
6	2.459459000	-0.418549000	-0.392609000
8	3.210713000	-1.152482000	-1.212584000
8	2.718343000	0.738322000	-0.150386000
1	1.522421000	-2.237868000	0.173126000
1	3.921664000	-0.591356000	-1.561761000

Table S12. Optimized geometry and Cartesian coordinates of PCA 4-OH···•C lipid model radical TS at M06-2X/6-311++G(d,p) level of theory in pentyl ethanoate.

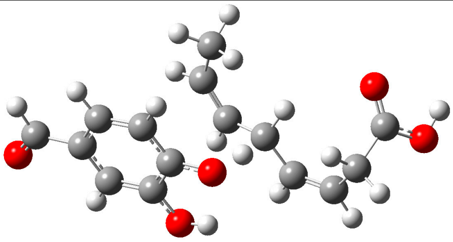

6	3.283567000	-1.515364000	-0.311936000
6	4.008661000	-0.756745000	0.775470000
6	2.112922000	-1.129845000	-0.834946000
6	1.353346000	0.069341000	-0.431833000
6	0.254086000	0.485904000	-1.297476000
6	-0.394081000	1.668448000	-1.220693000
6	-0.075733000	2.789248000	-0.285541000
1	3.765546000	-2.408838000	-0.693830000
1	4.667635000	-1.431433000	1.324401000
1	3.313030000	-0.293401000	1.474674000
1	1.661397000	-1.754726000	-1.603033000
1	1.960575000	0.882053000	-0.030796000
1	0.741102000	-0.278598000	0.613878000
1	-0.105509000	-0.246827000	-2.017171000
1	-1.214112000	1.836156000	-1.914427000
1	0.550890000	2.480982000	0.552151000
1	-0.996255000	3.225857000	0.109950000
6	4.842056000	0.340994000	0.159744000
8	4.580573000	1.515030000	0.191546000
8	5.920742000	-0.141490000	-0.476711000
1	6.385763000	0.603493000	-0.887833000
1	0.446435000	3.589824000	-0.822548000
6	-2.073383000	0.760793000	1.599166000
6	-3.343262000	0.964304000	1.099485000
6	-3.884267000	0.073447000	0.158460000
6	-3.165315000	-1.040796000	-0.277759000
6	-1.898623000	-1.259807000	0.230408000
6	-1.322066000	-0.359169000	1.182721000
1	-3.931213000	1.817787000	1.421256000
1	-3.593247000	-1.724358000	-1.002473000
6	-5.237996000	0.340863000	-0.369886000
8	-5.797535000	-0.340106000	-1.196625000
1	-5.730444000	1.238006000	0.049591000
8	-0.119327000	-0.619349000	1.607820000
1	-1.627612000	1.438514000	2.318101000
8	-1.171444000	-2.318662000	-0.164616000
1	-0.357540000	-2.327561000	0.364496000

Table S13. Optimized geometry and Cartesian coordinates of iron ion complexation with PCA in stoichiometric ratio 1:1 at M06/6-311++G(d,p) level of theory in water.

1:1 Fe^{2+}–PCA complex				1:1 Fe^{3+}–PCA complex			
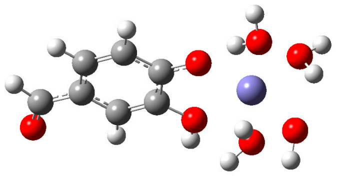				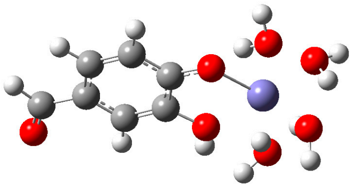			
26	1.956928000	-0.038413000	-0.005914000	26	-1.887395000	-0.021124000	-0.029300000
8	2.182061000	-0.016156000	2.235090000	8	-2.187319000	-0.124543000	-2.141053000
8	3.693065000	1.264470000	-0.197714000	8	-3.587094000	1.214145000	0.204059000
1	4.294697000	0.997407000	-0.902817000	1	-4.201321000	0.790164000	0.820058000
1	4.229355000	1.296383000	0.603457000	1	-4.065301000	1.291334000	-0.633409000
8	3.078156000	-1.898619000	0.072971000	8	-2.982033000	-1.827544000	0.193298000
1	2.943599000	-2.373464000	0.902257000	1	-3.041321000	-2.305899000	-0.645365000
1	2.823367000	-2.513629000	-0.625649000	1	-2.551294000	-2.433523000	0.812120000
8	1.952567000	-0.235920000	-2.249830000	8	-1.837188000	-0.216216000	2.159710000
1	1.917774000	0.649323000	-2.631876000	1	-1.743600000	0.676561000	2.519708000
1	1.126398000	-0.654809000	-2.519782000	1	-1.008272000	-0.662469000	2.382685000
1	1.427076000	-0.478092000	2.619081000	1	-1.439103000	-0.597529000	-2.531199000
1	2.091466000	0.897076000	2.532496000	1	-2.147021000	0.769460000	-2.508463000
6	-2.433743000	-0.804798000	0.031018000	6	2.366515000	-0.809804000	-0.077766000
6	-1.132739000	-0.386330000	0.041833000	6	1.071714000	-0.369806000	-0.106977000
6	-0.779897000	0.996738000	0.019223000	6	0.742324000	1.003459000	-0.050543000
6	-1.836375000	1.926422000	-0.002910000	6	1.776514000	1.939290000	0.033003000
6	-3.151189000	1.507220000	-0.010631000	6	3.087744000	1.504694000	0.063468000
6	-3.472187000	0.145393000	0.003288000	6	3.393073000	0.141806000	0.010492000
6	-4.856250000	-0.250567000	-0.008133000	6	4.788504000	-0.264988000	0.040412000
8	-0.057055000	-1.232403000	0.079285000	8	-0.034139000	-1.171568000	-0.208499000
8	0.469872000	1.346334000	0.022353000	8	-0.528875000	1.337006000	-0.080929000
1	-5.573450000	0.596254000	-0.025317000	1	5.509790000	0.574929000	0.105527000
1	-3.957721000	2.236847000	-0.028691000	1	3.899541000	2.224201000	0.131910000
1	-1.583638000	2.982743000	-0.015212000	1	1.523756000	2.993516000	0.077285000
1	-2.662890000	-1.868281000	0.045317000	1	2.592738000	-1.871580000	-0.123239000
1	-0.323310000	-2.160643000	0.060869000	1	0.146263000	-2.117975000	-0.109978000
8	-5.275987000	-1.404311000	0.000034000	8	5.186535000	-1.417516000	-0.002630000

Table S14. Optimized geometry and Cartesian coordinates of iron ion complexation with PCA in stoichiometric ratio 1:2 at M06/6-311++G(d,p) level of theory in water.

1:2 Fe^{2+}–PCA complex				1:2 Fe^{3+}–PCA complex			
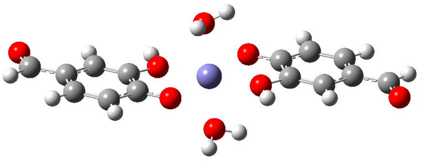				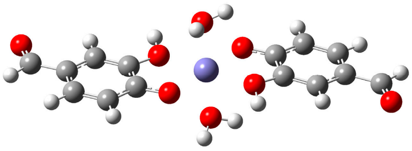			
26	-0.010835000	0.014803000	-0.026157000	26	-0.005339000	-0.012555000	0.075608000
8	0.233356000	0.013568000	-2.261548000	8	0.216432000	-0.026417000	-2.115046000
8	-0.258821000	0.017848000	2.210731000	8	-0.206753000	0.003812000	2.278491000
1	0.604298000	0.175577000	2.613428000	1	0.664445000	0.195047000	2.651587000
1	-0.499439000	-0.878139000	2.477288000	1	-0.417694000	-0.895158000	2.564067000
1	0.029320000	-0.864234000	-2.606933000	1	-0.005480000	-0.903473000	-2.454578000
1	1.165942000	0.154119000	-2.468611000	1	1.141702000	0.119635000	-2.354305000
6	4.292865000	-0.793773000	0.090044000	6	4.217551000	-0.790665000	0.042073000
6	3.010263000	-0.326868000	0.137294000	6	2.931629000	-0.335631000	0.125759000
6	2.702433000	1.068950000	0.110720000	6	2.610293000	1.043218000	0.092213000
6	3.795532000	1.956761000	0.039794000	6	3.662281000	1.961671000	-0.002117000
6	5.092020000	1.489804000	-0.006519000	6	4.966186000	1.512461000	-0.080347000
6	5.365229000	0.116912000	0.015134000	6	5.258073000	0.145416000	-0.067983000
6	6.732190000	-0.327529000	-0.032709000	6	6.641349000	-0.278539000	-0.157187000
8	1.905844000	-1.139698000	0.225817000	8	1.827975000	-1.146931000	0.278311000
8	1.472396000	1.468786000	0.151901000	8	1.349461000	1.401445000	0.148042000
1	7.476836000	0.493894000	-0.085825000	1	7.371436000	0.553779000	-0.226773000
1	5.923437000	2.189295000	-0.061570000	1	5.784440000	2.224225000	-0.156894000
1	3.581704000	3.021735000	0.021321000	1	3.424826000	3.020576000	-0.019568000
1	4.483760000	-1.864588000	0.112771000	1	4.428688000	-1.856555000	0.063939000
1	2.139594000	-2.076419000	0.197734000	1	1.991101000	-2.072388000	0.045557000
8	7.113458000	-1.495251000	-0.018385000	8	7.028789000	-1.437630000	-0.159450000
6	-4.305932000	0.804354000	-0.072090000	6	-4.222438000	0.824595000	-0.090621000
6	-3.024368000	0.338821000	-0.145842000	6	-2.941113000	0.350539000	-0.091087000
6	-2.713852000	-1.056275000	-0.123368000	6	-2.640693000	-1.033230000	-0.062335000
6	-3.803436000	-1.945484000	-0.024604000	6	-3.707075000	-1.938828000	-0.059008000
6	-5.099373000	-1.480101000	0.049108000	6	-5.007101000	-1.471165000	-0.064817000
6	-5.375064000	-0.107718000	0.028976000	6	-5.279387000	-0.100026000	-0.074541000
6	-6.741524000	0.334844000	0.101507000	6	-6.660341000	0.340245000	-0.071256000
8	-1.923615000	1.153080000	-0.263247000	8	-1.812288000	1.136414000	-0.123169000
8	-1.483800000	-1.452504000	-0.194582000	8	-1.382007000	-1.403662000	-0.043928000
1	-7.483674000	-0.487498000	0.172741000	1	-7.403152000	-0.483684000	-0.065960000
1	-5.928314000	-2.180652000	0.124241000	1	-5.838584000	-2.171665000	-0.058190000
1	-3.587976000	-3.010187000	-0.008492000	1	-3.486082000	-3.001322000	-0.045526000
1	-4.498544000	1.874813000	-0.094960000	1	-4.418603000	1.893442000	-0.103143000
1	-2.157627000	2.089696000	-0.236708000	1	-1.977904000	2.075382000	0.043483000
8	-7.125005000	1.501860000	0.089186000	8	-7.033557000	1.503998000	-0.073498000

Table S15. Optimized geometry and Cartesian coordinates of iron ion complexation with PCA in stoichiometric ratio 1:3 at M06/6-311++G(d,p) level of theory in water.

1:3 Fe^{2+}–PCA complex				1:3 Fe^{3+}–PCA complex			
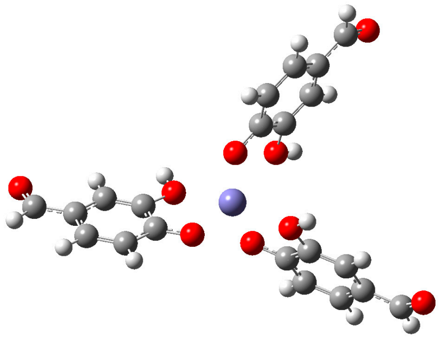				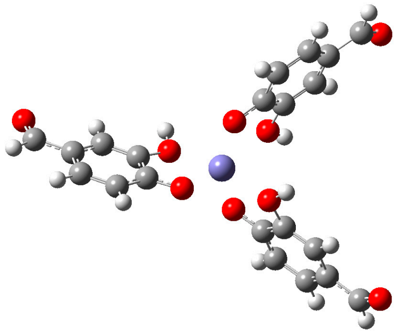			
26	-0.441024000	-0.244093000	0.000029000	26	-0.455039000	-0.200280000	0.009413000
6	-4.739723000	0.000046000	-0.792568000	6	-4.687285000	0.115506000	-0.780770000
6	-3.461308000	-0.262180000	-0.392678000	6	-3.406643000	-0.167710000	-0.400603000
6	-3.161661000	-0.875654000	0.864206000	6	-3.098947000	-0.815940000	0.819411000
6	-4.261071000	-1.204338000	1.685029000	6	-4.160960000	-1.195949000	1.647222000
6	-5.553504000	-0.939613000	1.286108000	6	-5.461487000	-0.916934000	1.271886000
6	-5.818579000	-0.336417000	0.050348000	6	-5.740994000	-0.259703000	0.070260000
8	-2.351600000	0.027481000	-1.151267000	8	-2.287645000	0.129078000	-1.146416000
8	-1.936169000	-1.102221000	1.203930000	8	-1.839245000	-1.021753000	1.122120000
1	-6.389145000	-1.197567000	1.933270000	1	-6.287964000	-1.202892000	1.917901000
1	-4.053883000	-1.670734000	2.644123000	1	-3.936503000	-1.695608000	2.584218000
1	-4.924259000	0.467210000	-1.757666000	1	-4.886524000	0.623022000	-1.720990000
1	-2.583632000	0.500173000	-1.961004000	1	-2.450655000	0.795344000	-1.828375000
6	2.460577000	3.190263000	-0.772906000	6	2.493129000	3.129140000	-0.804330000
6	1.465123000	2.292408000	-0.500184000	6	1.467749000	2.262717000	-0.531304000
6	0.601172000	2.446985000	0.626140000	6	0.694683000	2.377294000	0.651582000
6	0.803407000	3.576674000	1.441245000	6	0.986582000	3.419460000	1.538228000
6	1.804154000	4.484861000	1.165323000	6	2.019400000	4.296356000	1.266329000
6	2.648233000	4.307735000	0.063559000	6	2.782795000	4.159715000	0.104371000
8	1.198961000	1.194281000	-1.269656000	8	1.089019000	1.232764000	-1.342029000
8	-0.328318000	1.574943000	0.867171000	8	-0.268006000	1.513539000	0.880943000
1	1.950181000	5.351123000	1.806691000	1	2.252448000	5.100421000	1.959890000
1	0.147060000	3.706271000	2.297033000	1	0.391276000	3.508283000	2.441593000
1	3.101552000	3.041258000	-1.639429000	1	3.076257000	3.016183000	-1.715039000
1	1.851990000	1.101202000	-1.974803000	1	1.740314000	1.063930000	-2.035580000
6	3.293844000	-2.534878000	0.744686000	6	3.102944000	-2.614204000	0.769591000
6	2.085764000	-1.995594000	0.402676000	6	1.945589000	-2.000550000	0.383622000
6	1.390430000	-2.378882000	-0.785626000	6	1.316680000	-2.271612000	-0.856894000
6	2.002771000	-3.356360000	-1.594202000	6	1.915955000	-3.212454000	-1.703568000
6	3.220981000	-3.904958000	-1.250295000	6	3.086107000	-3.840608000	-1.321728000
6	3.886366000	-3.506098000	-0.085558000	6	3.692509000	-3.551917000	-0.095734000
8	1.426555000	-1.063041000	1.158042000	8	1.254509000	-1.086703000	1.140668000
8	0.249925000	-1.837704000	-1.080540000	8	0.208277000	-1.642692000	-1.153510000
1	3.681182000	-4.657742000	-1.886659000	1	3.552231000	-4.571233000	-1.978419000
1	1.485176000	-3.661985000	-2.499124000	1	1.441866000	-3.429389000	-2.655723000
1	3.792731000	-2.216579000	1.657514000	1	3.557633000	-2.383078000	1.729405000
1	1.956493000	-0.767098000	1.909024000	1	1.775846000	-0.710086000	1.863229000
6	3.682487000	5.275819000	-0.189868000	6	3.866790000	5.090025000	-0.136020000
8	4.491519000	5.243084000	-1.113409000	8	4.611195000	5.078248000	-1.106296000
1	3.715521000	6.110821000	0.540897000	1	3.994221000	5.859606000	0.653042000
6	-7.181761000	-0.081448000	-0.326114000	6	-7.119011000	0.022457000	-0.271599000
6	5.153812000	-4.104213000	0.239218000	6	4.915954000	-4.235798000	0.261010000
8	-7.559894000	0.431863000	-1.377135000	8	-7.498616000	0.594928000	-1.283858000
1	-7.929248000	-0.394969000	0.432440000	1	-7.856957000	-0.328822000	0.478850000
8	5.837598000	-3.853030000	1.228289000	8	5.545952000	-4.074337000	1.297860000
1	5.507209000	-4.855173000	-0.497713000	1	5.274776000	-4.958721000	-0.500390000

Table S16. Optimized geometry and Cartesian coordinates of iron ions in water at M06/6-311++G(d,p) level of theory.

$[Fe(H_2O)_6]^{2+}$				$[Fe(H_2O)_6]^{3+}$			
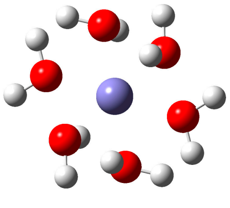				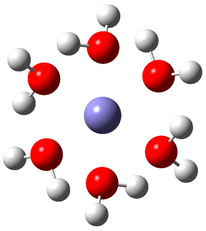			
26	0.000000000	0.000000000	0.000000000	26	0.000000000	0.000000000	0.000000000
8	-1.824913000	-0.516134000	-1.124891000	8	-1.705044000	-0.692187000	-1.029244000
8	1.193712000	-0.940927000	-1.603175000	8	1.195219000	-0.951489000	-1.531593000
1	2.138018000	-0.758932000	-1.525287000	1	2.029283000	-1.241226000	-1.133978000
1	1.118512000	-1.902485000	-1.584923000	1	0.749081000	-1.756620000	-1.830898000
8	-1.193712000	0.940927000	1.603175000	8	-1.195219000	0.951489000	1.531593000
1	-2.138018000	0.758932000	1.525287000	1	-2.029283000	1.241226000	1.133978000
1	-1.118512000	1.902485000	1.584923000	1	-0.749081000	1.756620000	1.830898000
8	-0.105964000	-1.898623000	1.096580000	8	0.008234000	-1.808909000	1.055593000
1	0.707502000	-2.409272000	1.000422000	1	0.856934000	-2.270169000	0.997466000
1	-0.204521000	-1.753639000	2.045580000	1	-0.184775000	-1.694226000	1.997018000
8	0.105964000	1.898623000	-1.096580000	8	-0.008234000	1.808909000	-1.055593000
1	0.204521000	1.753639000	-2.045580000	1	0.184775000	1.694226000	-1.997018000
1	-0.707502000	2.409272000	-1.000422000	1	-0.856934000	2.270169000	-0.997466000
8	1.824913000	0.516134000	1.124891000	8	1.705044000	0.692187000	1.029244000
1	1.654363000	0.483296000	2.074546000	1	1.740557000	0.248660000	1.890669000
1	2.069493000	1.432180000	0.943396000	1	1.615294000	1.637441000	1.220534000
1	-1.654363000	-0.483296000	-2.074546000	1	-1.740557000	-0.248660000	-1.890669000
1	-2.069493000	-1.432180000	-0.943396000	1	-1.615294000	-1.637441000	-1.220534000
